# A maladaptive pleural environment suppresses preexisting anti-tumor activity of pleural infiltrating T cells

**DOI:** 10.3389/fimmu.2023.1157697

**Published:** 2023-03-30

**Authors:** Vera S. Donnenberg, James D. Luketich, Ibrahim Sultan, John Lister, David L. Bartlett, Sohini Ghosh, Albert D. Donnenberg

**Affiliations:** ^1^ Department of Cardiothoracic Surgery, University of Pittsburgh School of Medicine, Pittsburgh, PA, United States; ^2^ University of Pittsburgh Medical Center (UPMC) Hillman Cancer Centers, Pittsburgh, PA, United States; ^3^ McGowan Institute for Regenerative Medicine, Pittsburgh, PA, United States; ^4^ Department of Medicine, Division of Hematology and Cellular Therapy, Allegheny Health Network Cancer Institute, Pittsburgh, PA, United States; ^5^ Drexel University College of Medicine, Philadelphia, PA, United States; ^6^ Department of Surgery, Division of Surgical Oncology, Allegheny Health Network Cancer Institute, Pittsburgh, PA, United States; ^7^ Department of Medicine, Division of Pulmonary and Critical Care Medicine, Allegheny Health Network, Pittsburgh, PA, United States; ^8^ Department of Medicine, University of Pittsburgh School of Medicine, Pittsburgh, PA, United States

**Keywords:** malignant pleural effusion, secretomics, immune checkpoint molecules, immunotherapy, adoptive cellular therapy, pleural T cells, Nanostring GeoMx

## Abstract

**Introduction:**

Treatment options for patients with malignant pleural effusions (MPE) are limited due, at least in part, to the unique environment of the pleural space, which drives an aggressive tumor state and governs the behavior of infiltrating immune cells. Modulation of the pleural environment may be a necessary step toward the development of effective treatments. We examine immune checkpoint molecule (ICM) expression on pleural T cells, the secretomes of pleural fluid, pleural infiltrating T cells (PIT), and ability to activate PIT *ex vivo.*

**Methods:**

ICM expression was determined on freshly drained and *in vitro* activated PIT from breast, lung and renal cell cancer. Secretomics (63 analytes) of activated PIT, primary tumor cultures and MPE fluid was determined using Luminex technology. Complementary digital spatial proteomic profiling (42 analytes) of CD45+ MPE cells was done using the Nanostring GeoMx platform. Cytolytic activity was measured against autologous tumor targets.

**Results:**

ICM expression was low on freshy isolated PIT; regulatory T cells (T-reg) were not detectable by GeoMx. *In vitro* activated PIT coexpressed PD-1, LAG-3 and TIGIT but were highly cytotoxic against autologous tumor and uniquely secreted cytokines and chemokines in the > 100 pM range. These included CCL4, CCL3, granzyme B, IL-13, TNFα, IL-2 IFNγ, GM-CSF, and perforin. Activated PIT also secreted high levels of IL-6, IL-8 and sIL-6Rα, which contribute to polarization of the pleural environment toward wound healing and the epithelial to mesenchymal transition. Addition of IL-6Rα antagonist to cultures reversed tumor EMT but did not alter PIT activation, cytokine secretion or cytotoxicity.

**Discussion:**

Despite the negative environment, immune effector cells are plentiful, persist in MPE in a quiescent state, and are easily activated and expanded in culture. Low expression of ICM on native PIT may explain reported lack of responsiveness to immune checkpoint blockade. The potent cytotoxic activity of activated PIT and a proof-of-concept clinical scale GMP-expansion experiment support their promise as a cellular therapeutic. We expect that a successful approach will require combining cellular therapy with pleural conditioning using immune checkpoint blockers together with inhibitors of upstream master cytokines such as the IL-6/IL-6R axis.

## Introduction

Breast and lung cancer, as well as a wide variety of other cancers, metastasize to the pleura (1). When they do, the outcome is uniformly fatal, with median survival measured in months (2). Routine drainage relieves symptoms of dyspnea and discomfort, but therapeutic efforts are often ineffective. The availability of drained MPE, which are discarded as medical waste, has opened a window onto the biology of metastasis, with ready access to tumor, infiltrating immune cells, as well as the cytokine-rich fluid that conditions the behavior of both populations. The mesothelial cells that line the parietal and visceral pleura are joined by tight junctions (3), forming a bioreactor-like space. In this space, cytokines and chemokines such as TGFβ1, IL-6, IL-6Rα and IL-8 (CXCL8) drive the tumor toward an aggressive mesenchymal state, while IL-10, IL-1RA and TGFβ1 actively suppress expression of immune effector functions (4). In this report we characterize pleural infiltrating T cells (PIT) for expression immune checkpoint molecules (ICM) and cytokine/chemokine secretion before and after *in vitro* activation, as well as their cytotoxic activity against autologous tumor.

## Materials and methods

### Patients and samples

Pleural effusions (PE) were collected as anonymized medical waste under an IRB exemption (No. 0503126), or IRB approved protocol No. 16110093, under which patients consented to use of the sample and access to medical records. Pleural effusions were collected fresh from 48 samples on 38 patients (21 breast cancer, 10 non-small cell lung cancer (NSCLC), 1 renal cell carcinoma, 1 cholangial carcinoma, 1 esophageal carcinoma, 4 normal physiologic pleural fluids). Normal pleural fluid (non-effusate) was obtained during cardiac surgeries (**Supplementary Table 1**). Peripheral blood mononuclear cells were prepared from heparinized peripheral blood and separated on a Ficoll-Hypaque gradient (5), (400 x g, 30 min, ambient temperature). Additionally, secretomic data were compared to 396 MPE cell-free fluid samples from our sample bank. These were obtained from a variety of cancers metastatic to the pleura, chiefly breast cancer (n=122), non-small cell lung cancer (n=61), and esophageal cancer (n=22).

### Secretomics

Cells were removed by centrifugation (10 min at 600 x g, 4°C), and then further clarified (10 min 1880 x g, 4°C) prior to storage at -86˚C. Immediately prior to analysis for cytokines and chemokines, samples were thawed and clarified by high-speed centrifugation (3 min at 16,000 x g, Beckman Microfuge E, Cat No. 348720, Beckman Coulter) in a cold room environment (4°C).

A total of 63 cytokines and chemokines were quantified on the Luminex platform (Hillman Cancer Center Luminex Core Facility), using the Curiox LT-MX plate washer, Curiox DA-96 plates, the Luminex 200 System analyzer and xPonent data acquisition and analysis software. Six-point standard curves were run for each cytokine with each of 2 sample batches. Cytokines were measured in 5 µL of neat, clarified effusion using the MILLIPLEX MAP Human Cytokine/Chemokine Magnetic Bead Panel - Premixed 38 Plex (Cat. No. HCYTMAG-60K-PX38), MILLIPLEX MAP Human TGFβ (Cat. No. TGFBMAG-64K-01), IL-6Rα from the Human Angiogenesis/Growth Factor Panel 2 (Cat. No. HANG2MAG-12K-01), MILLIPLEX MAP Human MMP Magnetic Bead Panel 2 (Cat. No. HMMP2MAG-55K), and MILLIPLEX^®^ Human Immuno-Oncology Checkpoint Protein Panel 2 (Cat. No. HCKP2-11K). Determinations that were designated “Out of Range Below” (*i.e.*, below the limit of quantification) by the analytical software were arbitrarily filled with a value 1/10 the lowest valid measurement for that cytokine. Values designated “Out of Range Above” (*i.e.*, above the limit of quantification) were assigned the value of the highest valid measurement for that cytokine.

### Proteomic profiling of CD45+ MPE cells

Cytocentrifuge preparations (Shandon Southern Cytospin) of fresh MPE cells, *in vitro* PIT and peripheral blood mononuclear cells were stained and analyzed using the Nanostring GeoMx^®^ Digital Spatial Profiling (DSP) platform (6) (Hillman Cancer Center Cytometry Core Facility). Immunofluorescent markers consisted of Pan-cytokeratin, CD45 and 4′,6-diamidino-2-phenylindole (DAPI). The protein panel consisted of 6 control antibodies, plus 42 antibodies from the human protein core, immuno-oncology (IO) drug target, immune activation status, immune cell typing, and myeloid panels. Slides were processed per manufacturer’s instructions and regions of interest (ROI) rich in CD45+ were identified. Antibody barcodes were counted on the Ncounter^®^ platform per manufacturer’s instructions and QC performed in the DSP analysis suite prior to data analysis.

### Small-scale T-cell *in vitro* activation and tumor cell expansion

Freshly drained pleural fluid was from breast cancer patients were plated in T75 culture flasks (10^7^ cells/flask) in Mammary Epithelial Cell Growth Medium (MEGM, Lonza, Cat. No. CC-3150) supplemented with 10% cell-free MPE fluid, penicillin (100U/mL) and gentamicin (50μg/mL), and cultured 24-48 h (37°C, 5% CO_2_ in air), after which a T-cell enriched non-adherent fraction was collected. Effusions for all other cancers were plated in Bronchial Epithelial Growth Medium (BEGM, Lonza, Cat. No. CC-3170). The adherent fraction was refluided with MEGM or BEGM and allowed to grow to 70-80% confluence (passage 0). Supernatants were removed for secretomic analysis and adherent cells were trypsinized (0.25% trypsin, 2.2 mM EDTA, Corning Cat. No. 25-053-CI). After removing an aliquot for flow cytometry, tumor cells were seeded onto 96 well round-bottom plates for the cytotoxicity assay (passage 1). Autologous tumor cells no later than passage 2 were used as targets in cellular cytotoxicity assays (**Supplementary Figure 1**). Target cells expressed markers consistent with partial epithelial to mesenchymal transition (EMT) and typical of aggressive disease (7, 8). An independent experiment determining the effects of IL-6Rα antagonist, tocilizumab, on the epithelial to mesenchymal transition and epithelial state of cultured tumor targets was performed by co-culturing tumor cells in the presence of 3µg/ml (0.02069 µM) of tocilizumab (Genentech MTA00004791) for the duration of culture expansion. The effects of IL-6Rα inhibition during pleural T-cell activation were also determined, using graded doses of tocilizumab during culture expansion in triplicate (0, 0.0026, 0.0052, 0.010, 0.021, 0.041 µM; for 7 or 10 days).

The non-adherent T-cell enriched fraction was cultured in 12 x 75mm snap cap tubes (2 x 10^6^ cells in 2 mL) in RPMI-1640 medium supplemented with 10% human AB serum, L-glutamine (200μM), 2-mercaptoethanol (0.05mM), penicillin (100U/mL), gentamicin (50μg/mL) and HEPES buffer (2.5 mM). Non-stimulated cultures received medium alone; stimulated cultures also received CD3/CD28 beads (Gibco Cat. No. 11131D, 1:1 with cells) and IL-2 (180U/mL), unless otherwise noted. T-cell cultures were split when they reached approximately 2 x 10^6^ cells/mL.

### Large-scale cGMP expansion of pleural T cells

Pleural T cells were separated and expanded using the Miltenyi CliniMACS Prodigy system by a protocol similar to that developed for CAR-T expansion (9). GMP-compliant materials (Miltenyi Biotech, Bergisch Gladbach, Germany, TS520 tubing set) and reagents were used throughout. After filtration through a 170 - 260 μm blood filter (Baxter, Deerfield IL, Cat. No. 2C8750s), CD4+ and CD8+ cells were immunomagnetically separated using CliniMACS GMP CD4 (Cat. No. 170-076-702) and CliniMACS GMP CD8 (Cat. No. 170-076-703) microbead reagents. Cells were activated with MACS GMP T Cell TransAct (Miltenyi, Cat. No. 130-111-160), a colloidal polymeric nanomatrix containing anti-CD3 and anti-CD28. Non-target cells were collected and expanded for use as cytotoxicity targets. After washing out TransAct, cells were cultured in TexMACs medium (Miltenyi, Cat. No. 170-76-306) supplemented with 3% human AB serum (Innovative Research, Novi, MI, Cat. No. ISERABHI-100), penicillin G (100 U/mL, MP Biomedicals 194537) and Gentamicin sulfate (50 µg/mL, Millipore Sigma, G1264-5G), IL-7 (12.5 ng/mL, Miltenyi, Cat. No. 76111) and IL-15 (12.5 ng/mL, Cat. No. 76114 Miltenyi Biotech). Cells were cultured for 8 days in a temperature (37°C) and CO_2_ (5%) controlled chamber with continuous medium exchange.

### Cellular cytotoxicity

Twenty-four hours prior to assay, autologous tumor cell primary cultures were trypsinized and plated in basal MEGM (breast cancer) or BEGM (NSCLC, RCC) medium (without addition of the growth factor bullet kit) at 10,000 cells/well in 96-well round-bottom plates (target cells). The activated, expanded T-cell enriched fraction (effector cells) was washed, counted, and resuspended in RPMI-1640 complete medium. CD3/CD28 beads were removed with a strong magnet according to the manufacturer’s instructions. After carefully removing culture medium from the target cell wells for secretomic analysis, effector cells were added at varying effector to target ratios (0:1, 1:1, 3:1, 6:1, 12:1, 25:1, 50:1) in triplicate (total culture volume ~120 μL). Additionally, in selected experiments PIT alone were plated at concentrations corresponding to the E:T ratios (500,000, 250,000, 125,000, 6,000, 3,000 and 1,000 cells per well). Plates were then centrifuged (200 x g, 10 min) and cultured for 4h (37°C, 5% CO_2_ in air), and 50μL of supernatant was transferred to a flat bottom 96-well plate for analysis of lactate dehydrogenase (LDH) release (Promega CytoTox 96 Non-Radioactive Cytotoxicity Assay, Cat. No. G1780). Maximal LDH release was determined by adding the provided lyse solution to target cells. Spontaneous release was measured in target cell supernatant plated in the absence of effector cells. After addition of the provided tetrazolium salt substate, the concentration of released LDH (determined by the conversion of the substrate into a red formazan product) was measured by absorbance at 490nm. Specific lysis (SL) was determined according to the formula:


$$SL\%=\left(\;\frac{OD_{Experimental}\;-\;OD_{Spontaneous}}{OD_{Maxiumal}-\;OD_{Spontaneous}}\right)\times 100$$

Where OD is the optical density.

The cytolytic index was determined according to the method of Henney (10) from the slope of the least squares line of best fit of SL% versus effector to target cell (E:T) ratio x 1000.

After supernatant harvest, the remaining effector cells (E:T 12, 25, 50) were removed, pooled, washed and stained for expression of CD45, CD3, CD4, CD8, effector molecules (perforin, granzyme B), and PD-1 (**Supplementary Figures 2A, B**).

### Flow cytometry

Sample preparation and flow cytometry for cell-surface and intracellular markers was performed as previously described in detail (11). Freshly isolated MPE cells, *in vitro* activated non-adherent cells or trypsinized cultured MPE tumor cells were washed in staining buffer consisting of 4% calf serum, 2 mM EDTA in PBS without Ca+2 and Mg+2 (pH 7.2). Supernatant was decanted and the cell pellet incubated with neat decomplemented mouse serum (10 µL, 5 min, 4°C) and then stained for cell surface markers with 2 µL each of antibodies directed against cell-surface markers (20 min, 4°C). A complete list of antibody reagents, vendors and catalog numbers is provided in **Supplementary Table 2**.

Cells were pelleted and fixed with 2% methanol-free formaldehyde (Polysciences, Warrington PA, Cat. No. 0401A) for 20 min, permeabilized with 0.05% Saponin (Coulter) in staining buffer and washed with staining buffer. Cells were then stained with 2μL of antibodies directed against intracellular antigens (30 min, ambient temperature), washed in staining buffer, and resuspended in staining buffer containing PureLink RNAse A (Invitrogen, Cat. No. 12091-021, 1:400, 10 min 37°C) and washed. Finally, cells were resuspended in staining buffer to a concentration of 5 x 10^6^/mL and DAPI (Invitrogen Cat. No. D1306) was added to the cells at a concentration of 10 μg/mL for determination of DNA content.

Cells were acquired on a Fortessa SORP flow cytometer (BD Biosciences, San Diego, CA), calibrated daily with CS and T beads (BD Biosciences, Cat. No. 650621). PMT voltages were adjusted to predetermined target channels using the seventh peak of 8-peak Rainbow Calibration Particles (Spherotech, Lake Forest, IL, Cat. No. RCP-30-5A); as a reference point. FITC, PE (BD Biosciences, Cat. No. 349502) and APC (BD Biosciences, Cat. No. 340487) Calibrite beads, single stained BD CompBeads anti-mouse IGκ (for all tandem fluorochromes; BD Biosciences Cat. No. 51-90-9001229), and unstained cells (DAPI only) were used as spectral compensation standards. Acquired data were analyzed using VenturiOne Software V7.3 (Applied Cytometry Systems, Dinnington, UK).

### Statistical analysis

ANOVA was performed on ICM expression (**Supplementary Table 3**). Equal variances were assumed, and Tukey’s honestly significant difference test was used for between group comparisons. Secretomic analysis was performed on log_10_-transformed data. SYSTAT 13 software (San Jose, CA) was used for data analysis and graphics. Nanostring GeoMx proteomic data (Ncounter counts) were normalized to GAPDH counts. Data were displayed as a heatmap of log-transformed data with Pearson -1 hierarchical clustering using the Morpheus utility (Broad Institute, https://software.broadinstitute.org/morpheus/).

## Results

### Freshly isolated PIT are not exhausted

**Figure 1:**
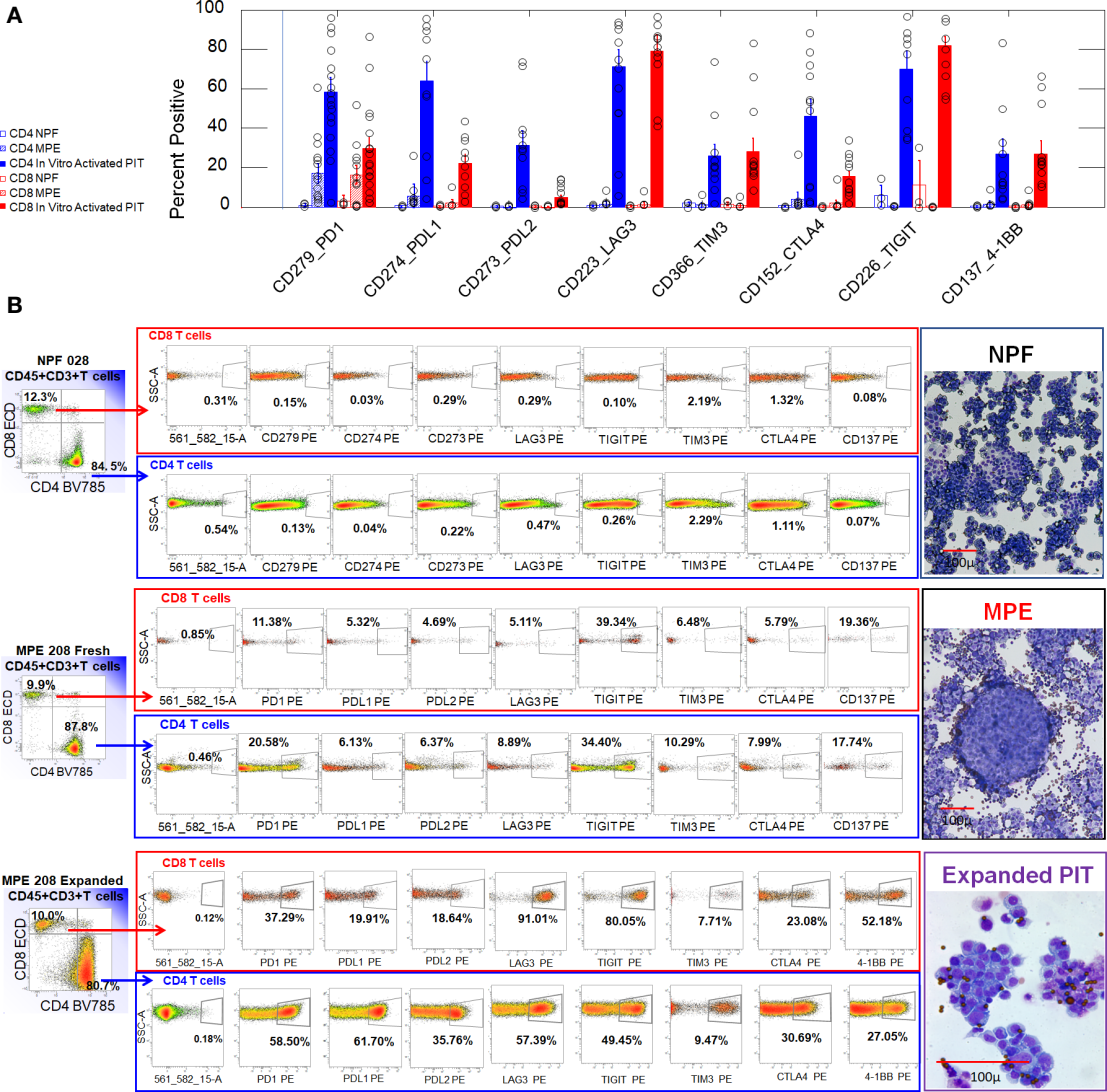
Expression of ICM on CD4+ and CD8+ T cells from normal pleural fluid, freshly drained MPE and *in vitro* activated non-adherent MPE cells. **(A)** Bar graph summarizing results from 3 NPF (2 valve replacement, 1 aortic aneurism), 13 MPE (serial samples from MPE205 Breast Cancer (ER+PR+Her2+), MPE208 Breast Cancer (ER+PR-), MPE212 Breast Cancer (ER+PR+Her2-), MPE211 Renal Cell Carcinoma), and 17 *in vitro* activated PIT (same subjects). Error bars indicate standard errors of the means. **(B)** Representative histograms used to generate the bar graph shown in Panel **(A)**. The parent gate for CD4 and CD8 T cells was CD45+, CD3+ singlet cells (not shown). Illustrative cytocentrifuge preparations of freshly isolated normal pleural fluid (NPF) and a breast cancer malignant pleural effusion (MPE), and *in vitro* activated PIT (Wright Giemsa stain) are shown to the right of the histograms. The smallest dark circles in NPF and MPE are lymphocytes. The sheets of large cells in NPF are shed mesothelial cells. The large cluster of cells in MPE is a tumor cell nest. *In vitro* activated PIT are characterized by proliferating cell clusters and are shown at a higher magnification: the small dark circles are CD3/CD28 beads.

We examined expression of the negative regulatory ICM PD-1, PD-L1, PD-L2 LAG-3, TIM-3, CTLA-4 and TIGIT, and the costimulatory IC molecule 4-1BB on CD4+ and CD8+ T cells from freshly isolated normal pleural fluid (NPF), malignant pleural effusions (MPE), and *in vitro* activated non-adherent cells isolated from MPE (**Figure 1**). Freshly isolated NPF and MPE T cells had uniformly low expression of ICM. A proportion of CD4 (17 ± 4.9%, mean, SEM) and CD8+ (16.5 ± 4.5%) MPE T cells expressed PD-1. In contrast, *in vitro* activated CD4+ and CD8+ T cells had robust expression of PD-1 (53.1 ± 7.7% and 27.3 ± 5.5%, respectively), LAG-3 (64.8 ± 9.0% and 74.3 ± 7.9%) and TIGIT (67.2 ± 8.3% and 77.3 ± 5.4%). Activated CD4+ T cells also had elevated levels of PD-L1 (59.5 ± 10.6%) and PD-L2 (30.8 ± 7.4%). ANOVA contrasts of ICM on all sample types are shown in **Supplementary Table 3**.

### Secretomic analysis of *in vitro* activated PIT

**Figure 2:**
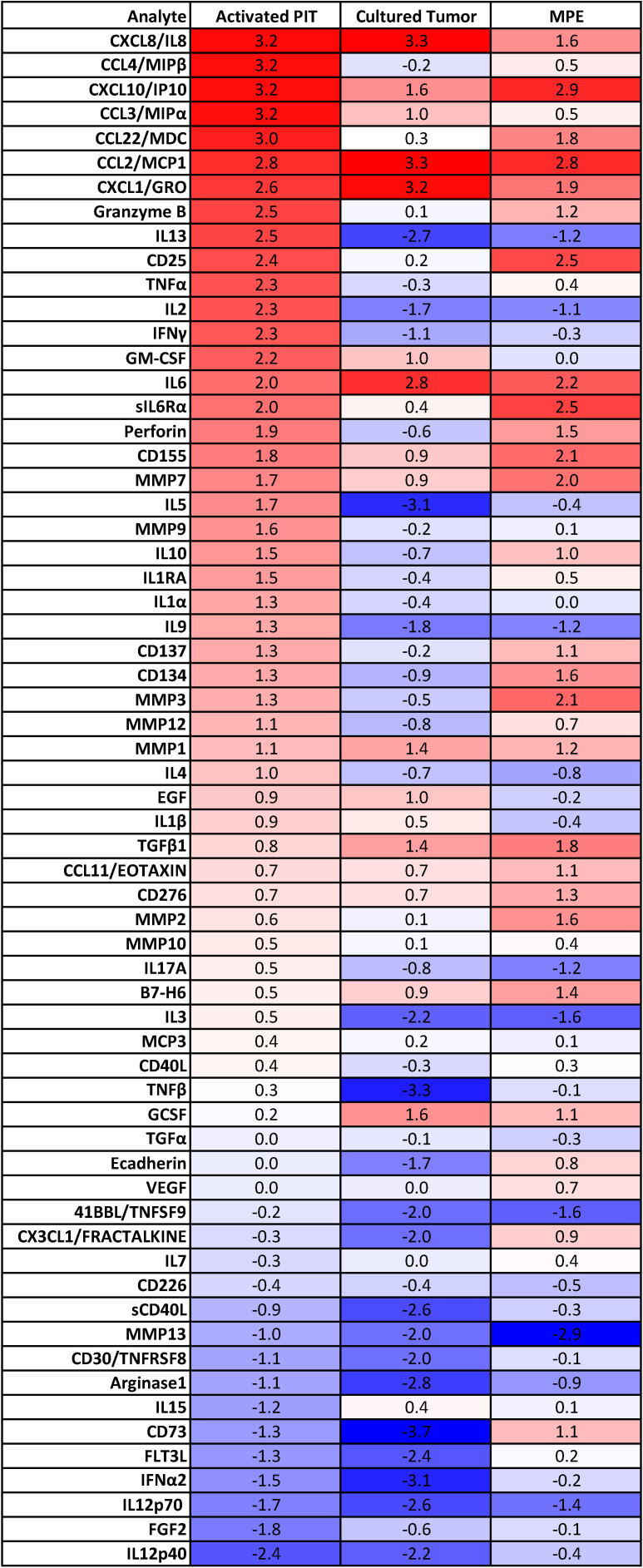
Secretome of culture supernatants of *in vitro* activated PIT (n=10), primary MPE tumor cell cultures (n=39) and MPE cell-free fluid (n=396). Data within heatmap boxes are expressed as log_10_ picomolar mean concentrations because of the disparity in cytokine and chemokine molecular masses, where bright red indicates approximately 2000 pM, white indicates 2 pM and blue indicates 0.002 pM. Cytokine and chemokine molecular masses were determined from the literature and do not account for isoforms, glycosylation or other sources of variability.

Culture supernatants from *in vitro* activated and expanded non-adherent MPE cells (IL-2, CD3/CD28 beads, n = 10) were harvested between 7 and 21 days after culture initiation and assayed for the presence of 63 cytokines and chemokines using Luminex technology. We compared the secretome of *in vitro* activated non-adherent MPE cells (activated PIT) with that of cultured MPE tumor cells (n = 35), and with our database of MPE cell-free fluids (n=396) from a variety of cancers. Data were expressed as a heatmap in **Figure 2** and ordered (top to bottom) by their pM concentration in *in vitro* activated PIT supernatants. For comparison with existing literature, the same data are shown as pg/mL (**Supplementary Figure 5**).

Comparing activated PIT and Cultured Tumor, we can see which cytokines/chemokines they have in common, and which are unique. Numerous cytokines and chemokines are elevated in activated PIT culture supernatants (10 - 1500 pM), compared to cultured tumor (**Figure 2**). In order of concentration, these are: MIP1β (CCL4), MIP1α (CCL3), CCL22/MDC, granzyme B, IL-13, CD25, TNFα, IL-2, IFNγ, GM-CSF, IL-6Rα, perforin, CD155, MMP7, IL-5, MMP9, IL-10, IL-1RA, IL-1α, IL-9, CD137, CD134, MMP3, MMP12 and IL-4. The presence of MMPs indicates that macrophages are also present in the activated PIT cultures. Photomicrographs documenting macrophages associated with lymphocyte clusters during *in vitro* activation are shown in **Supplementary Figure 3**.

Comparing activated PIT to fresh MPE cell-free fluid (CFF), we can infer which immune cell associated cytokines and chemokines are upregulated when PIT and antigen presenting cells are removed from the pleural environment and activated *ex vivo*: MIP1β (CCL4), MIP1α (CCL3), granzyme B, IL-13, TNFα, IL-2, IFNγ, GM-CSF, IL-5, MMP9, IL-1RA, IL-1α, IL-9 and IL-4.

Low passage tumor alone secreted high concentrations of CCL2/MCP1, CXCL8/IL-8, CXCL1/GRO, IL-6, CXCL10/IP10, G-CSF, TGFβ1, MMP1, CCL3/MIPα, GM-CSF and EGF.

**Figure 3:**
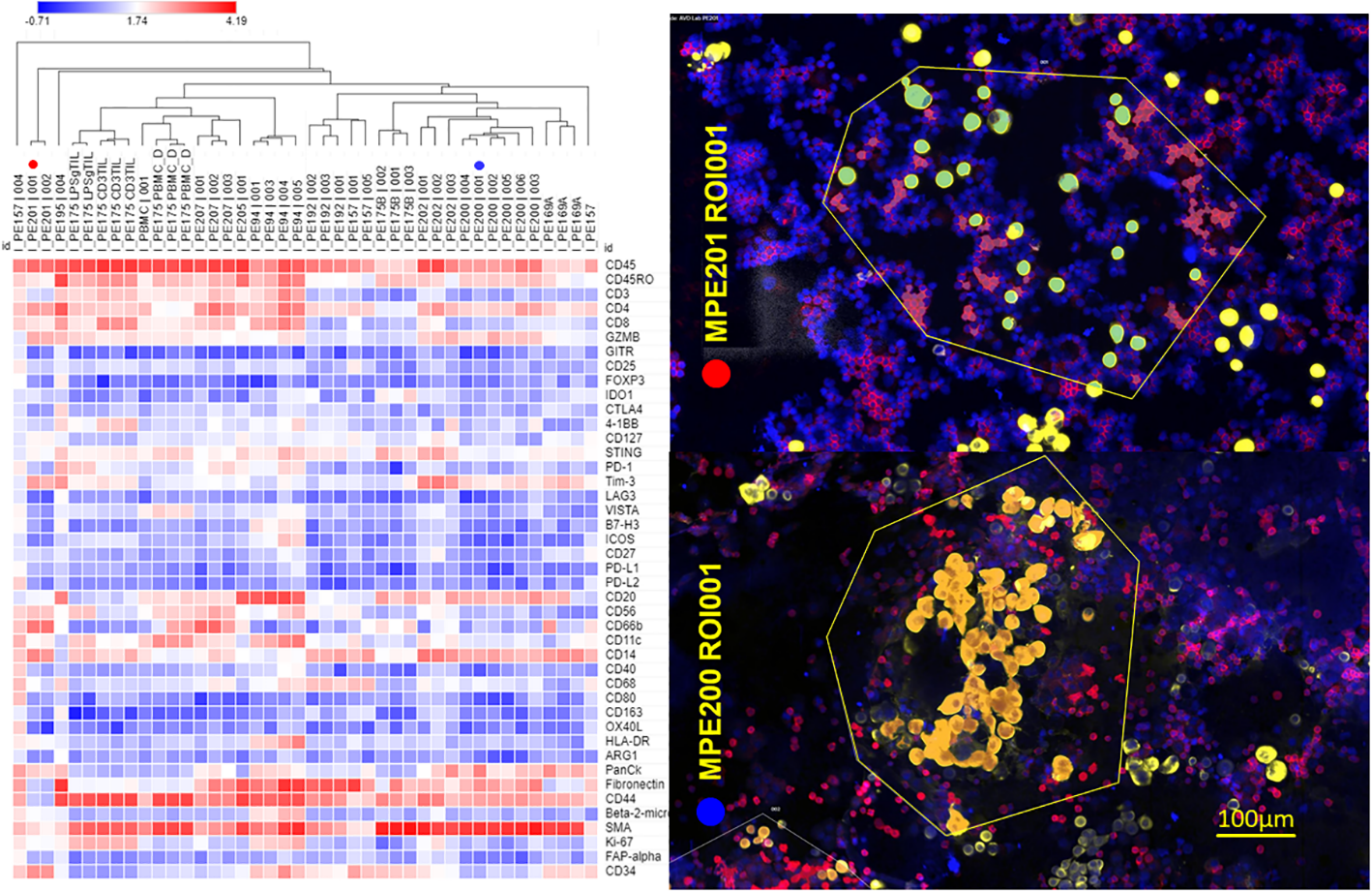
Proteomic profiling of CD45+ MPE cells. NanoString GeoMx digital spatial profiling was performed on 12 breast cancer MPE cytocentrifuge preparations, 2 peripheral blood mononuclear cell preparations (PBMC), and one activated PIT. Left panel: Regions of interest (ROI) were segmented on CD45 positivity determined by immunofluorescence. Log-transformed normalized data are displayed as a heatmap with hierarchical clustering of the samples/ROI. Sample identification number and ROI appear above each column; CD3 TIL = CD3/CD28/IL-2 activated, LPSg = LPS/IFNγ activated, PBMC = freshly isolated peripheral blood mononuclear cells. PE201 (ROI 1, red circle) and PE200 (ROI 1, blue circle) represent T-cell-rich and macrophage-rich samples, respectively. Right panel: False-colored immunofluorescent photomicrographs (PE201/ROI 1, PE200 ROI 1) used for selecting ROI (enclosed in yellow polygons). Red = CD45+, Gold = pan-cytokeratin+, Blue DAPI+. Proteomic analysis was performed on CD45+ (red) segments within ROI.

*Proteomic analysis of CD45+ MPE cells.* Cytocentrifuge slides were prepared from 12 breast cancer MPE, two peripheral blood mononuclear cell preparations (PBMC), and one activated PIT (2 conditions: IL-2/CD3/CD28/IFNγ and LPS/IFNγ). Data (43 analytes, GAPDH-normalized log-transformed Ncounter counts) are displayed as heatmaps (**Figure 3**) with hierarchical clustering of samples (columns). Regions of interest (ROI) were segmented on CD45 expression determined by immunofluorescence (**Figure 3**, right panel). Regulatory T cells (FOXP3+, CD25+, GITR+) were not detected, and ICM (PD-1, PD-L1, PD-L2, LAG3, CTLA-4, 4-1BB, VISTA, B7-H3) expression was low in all MPE. Samples clustering toward the right have fewer CD3+ T cells, CD8+ cells, fewer proliferating Ki-67+ cells, more macrophages and higher Tim-3 expression. Regions of interest showed greater similarity within samples than between samples. CD45RO+ CD27-effector-memory T cells (12–15) and CD20+ B cells were prominent in most samples. Detection of cytokeratin and CD34 in some samples indicates the presence of epithelial and endothelial cells in some CD45+ fields. PE201 (ROI 1, red circle) and PE200 (ROI 1, blue circle) represent T-cell-rich and macrophage-rich samples, respectively (**Figure 3**, right panel).

### Antitumor activity of activated pleural T cells

**Figure 4:**
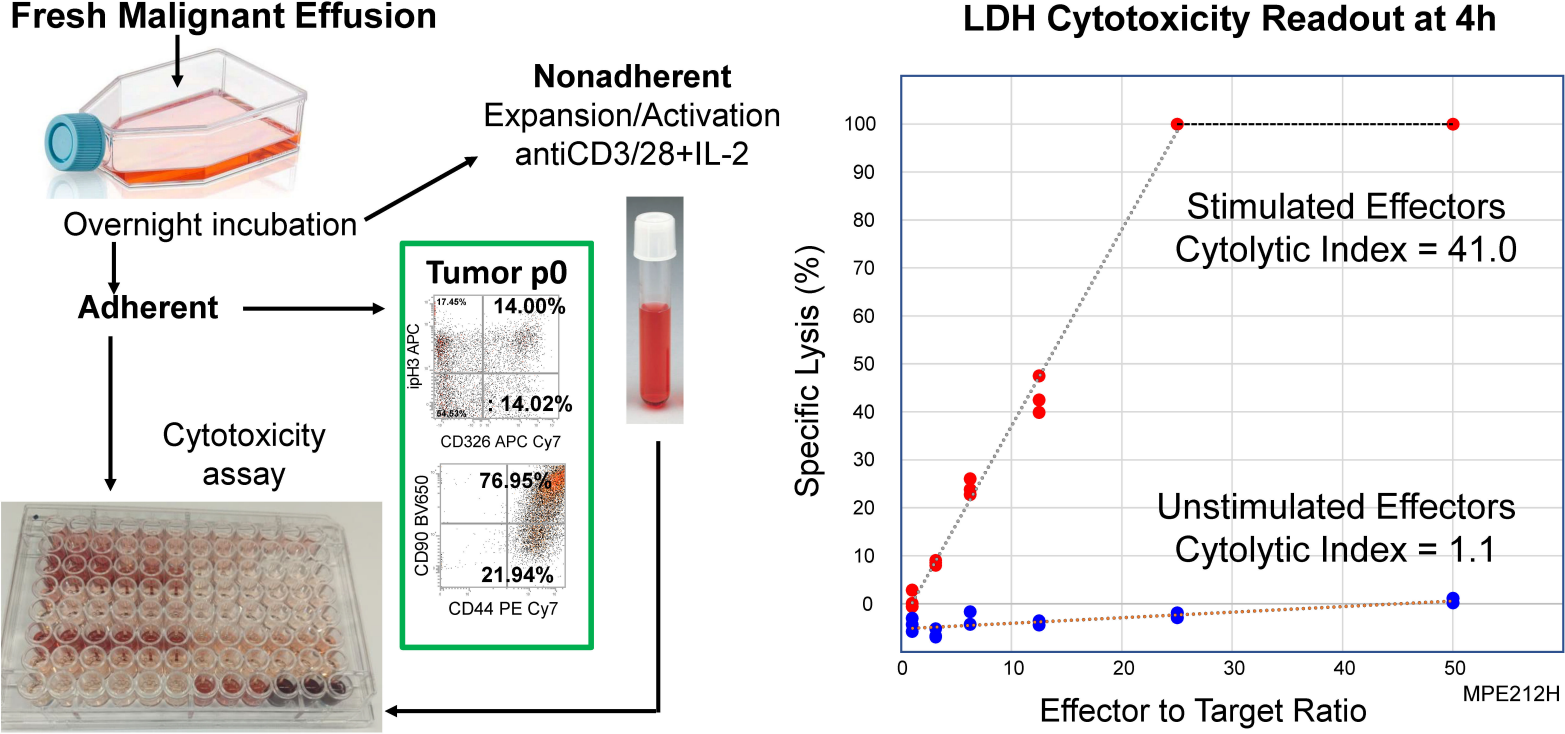
Activated PIT are cytolytic to autologous tumor. A T-cell enriched fraction was obtained from a freshly drained MPE (PE212H, **Table 1**) as non-adherent cells after overnight culture in a polystyrene flask. The T-cell enriched fraction was cultured in the presence (Stimulated) or absence (Unstimulated) of CD3/CD28 beads plus IL-2 for 14 days, after which time they were co-incubated with autologous tumor. Cytotoxicity was measured by LDH release after 4 hours in culture. Left Panel: Schematic of cytotoxicity assay. Right Panel: Cytotoxic activity was quantified as the cytolytic index, determined from the slope of the kill curve x 1000.

**Figure 4** shows the schema for assessing anti-tumor cytotoxic activity of *in vitro* activated PIT against autologous tumor, and a representative example, showing determination of the cytolytic index from the slope of the kill curve. Photomicrographs documenting tumor cytolysis at high E:T ratios, and flow cytometric characterization of the effector cells following 4h exposure to autologous tumor are shown in **Supplementary Figure 2**. Stimulated CD4+ effector cells dimly coexpressed CD8. These double positive cells had high coexpression of granzyme B and perforin. CD8+ T cells had high expression of PD-1 (CD279) compared to CD4. Unstimulated (mock effector) pleural T cells lacked CD4+ CD8^dim^ cells, did not express granzyme B or perforin, but upregulated PD-1. In the absence of *ex vivo* stimulation, cultured non-adherent MPE cells did not kill autologous tumor. The results from seven patients, one of whom (PE212) was drained on multiple occasions, are shown in **Table 1**.

**Table 1:** Cytotoxicity of *in vitro* activated, culture expanded MPE T cells against autologous tumor.

Cytolytic activity of *in vitro* activated PIT					
Sample	Dx	Stimulus	Expansion Time (Days)	Cytolytic Index	Intercurrent Treatment
MPE211	RCCa	CD3/CD28/IL2	21	12.1	Ipilimumab/Nivolumab
MPE208A	BrCa ER+PR-	CD3/CD28/IL2	24	78.5	Untreated
MPE212B	BrCa ER+ PR+ Her2-	CD3/CD28/IL2	21	4.2	Capecitabine
MPE212B	BrCa ER+ PR+ Her2-	LPS/IFNγ	21	6.2	Capecitabine
MPE212B	BrCa ER+ PR+ Her2-	CD3/CD28/IL2/IFNγ	21	7.1	Capecitabine
MPE212H	BrCa ER+ PR+ Her2-	CD3/CD28/IL2	7	13.7	Capecitabine
MPE212H	BrCa ER+ PR+ Her2-	CD3/CD28/IL2	14	41.0	Capecitabine
MPE212O	BrCa ER+ PR+ Her2-	CD3/CD28/IL2	4	47.3	Off Chemo (2 weeks)
MPE212P	BrCa ER+ PR+ Her2-	CD3/CD28/IL2	2	42.0	Off Chemo (4 weeks)
MPE163	NSCLC	CD3/CD28/IL7+IL15	5	17.7	Unknown
MPE173	NSCLC	CD3/CD28/IL7+IL15	5	12.3	Untreated
MPE213	NSCLC	CD3/CD28/IL2	2	14.4	Osimertinib
MPE213	NSCLC	CD3/CD28/IL7+IL15	2	16.4	Osimertinib
MPE213	NSCLC	CD3/CD28/IL2	7	17.1	Osimertinib
MPE213	NSCLC	CD3/CD28/IL7+IL15	7	16.6	Osimertinib
MPE215*	NSCLC	CD3/CD28/IL7+IL15	7	4.5	Untreated
*MPE215 was a clinical-scale expansion.			Mean	21.9	
			SD	20.2	

MPE from two breast cancer patients, one patient with renal cell carcinoma (RCC) and 4 patients with non-small cell lung carcinoma were studied. Cells drained from patient PE212 was studied on multiple occasions as indicated by the letter suffixes. PE215 was expanded on a clinical scale using the CliniMACS Prodigy system.

In initial experiments, the activating stimulus was CD3/CD28 beads plus IL-2, but other strategies were tested. In one instance IFNγ (500 U/mL) was added, and in another, cells were stimulated with bacterial lipopolysaccharide (LPS, 10 ng/mL) plus IFNγ. Finally, based on recent findings in CAR-T expansion, we settled on CD3/CD28 beads and IL-7 plus IL-15 (16).

Robust killing was observed in all cases. Activated cells from Subject PE212, who was studied on seven separate occasions, killed autologous tumor even when the patient was receiving the immunosuppressive drug capecitabine (17), but killing improved markedly when the drug was discontinued. Impressive killing was noted even when the cells were activated for only two days, a time insufficient for significant expansion. In the case of PE215, immunomagnetically selected CD4+ and CD8+ PIT were expanded on a clinical scale. In one experiment, no killing was observed when autologous adherent peripheral blood monocytes were substituted for tumor targets (data not shown). In a separate experiment, in which unstimulated freshly isolated sort-purified CD3+ T cells were plated on autologous tumor targets from a prior MPE drainage, no killing was observed (data not shown).

### Effect of IL-6Rα antagonism on tumor EMT

**Figure 5:**
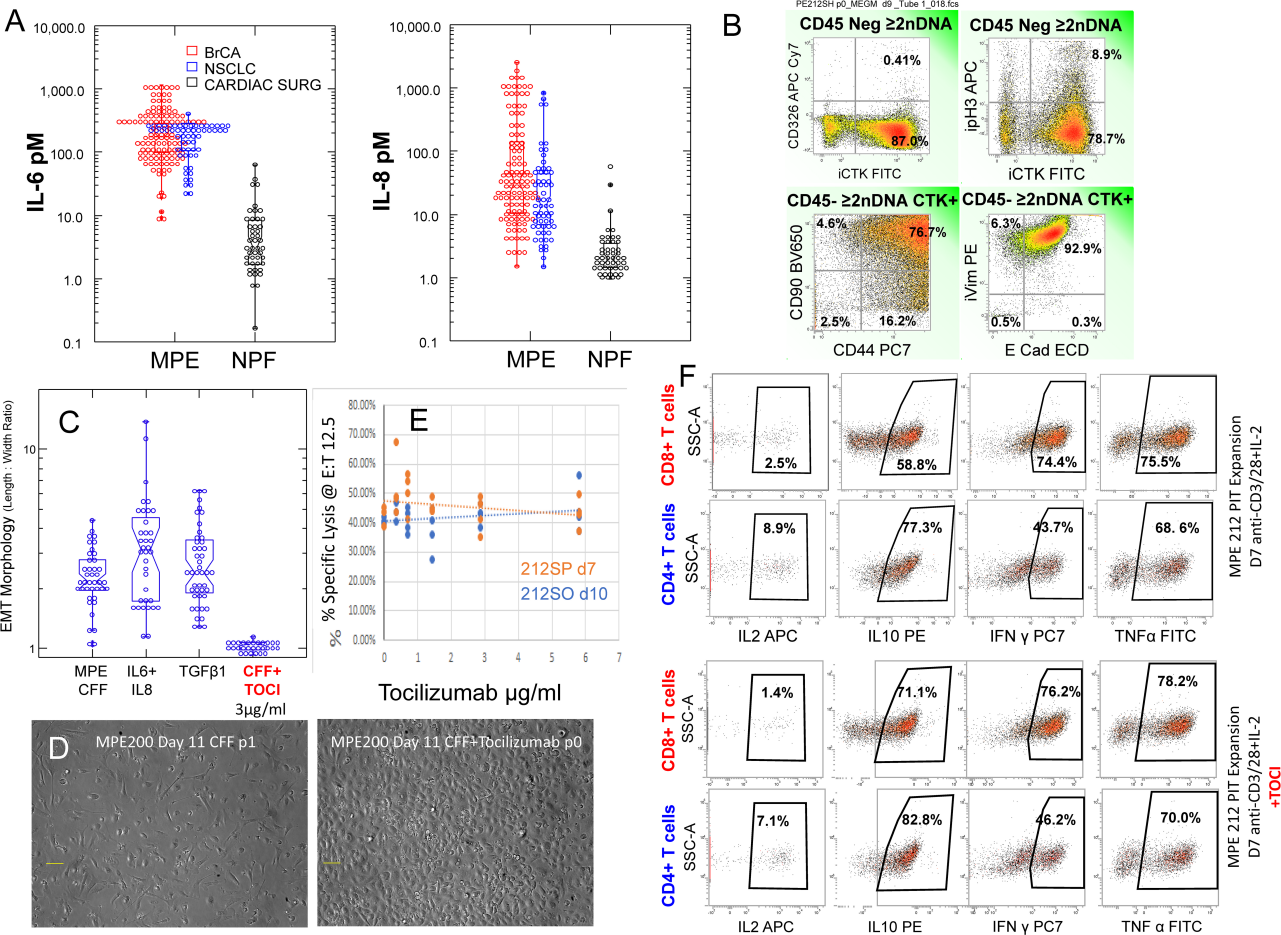
Effects of Tocilizumab on tumor EMT, antitumor cytotoxic effector function and cytokine secretion. **(A)** IL-6 and IL-8 levels in MPE fluid from Breast cancer (red circles, all subtypes, n = 122) and non-small cell lung cancer (blue circles, n = 61) compared to normal pleural fluid isolated from patients undergoing cardiac surgery (black circles). **(B)** Flow cytometry on passage 0 MPE tumor cells. Histograms are gated in CD45 negative aneuploid (>2N DNA content) cells. The majority of cytokeratin+ cells have lost surface expression of CD326 (EpCAM), coexpress CD90, CD44, vimentin and E-cadherin, indicating pEMT. 10.2% of cytokeratin+ tumor cells are in cycle as indicated by histone H3 phosphorylation (pH3). **(C, D)** The majority of MPE tumor cells cultured in the presence of autologous cell free fluid (CFF) or medium supplemented with IL-6, IL-8 or TGFβ demonstrate a fibroblastoid morphology quantified by the length to width ratio (left panel). Addition of tocilizumab (3µg/mL) to cultures containing CFF blocked EMT, causing cells to transition to epithelioid morphology **(D)** right panel. **(E)** Pleural T cells, activated and expanded in the presence of CD3/CD28 beads plus IL-2 are potent cytotoxic effectors against autologous tumor as measured by LDH release at a fixed effector to target ratio of 12.5: 1. Addition of graded doses of tocilizumab during activation and expansion did not alter cytotoxic activity. **(F)** The same cells that were tested for cytolytic activity **(D)** were evaluated for cytokine production by flow cytometry with (pooled 3 and 6 μg/mL) and without tocilizumab. Activated CD4 and CD8 pleural T cells actively produced IL-10, IFNγ and TNFα. Cytokine production was not affected by inclusion of tocilizumab during activation.

The secretome of the malignant pleural environment is dominated by IL-6 and IL-8/CXCL8, cytokines that are known to promote EMT and which are virtually absent from normal non-effusate pleural fluid from age-matched patients (**Figure 5A**). Low passage cytokeratin+, aneuploid MPE tumor cells evidenced a high degree of EMT as documented by surface expression of CD90, CD44, and loss of surface expression of EpCAM (**Figure 5B**). High coexpression of CD44, CD90 and vimentin and spindle shaped morphology (**Supplementary Figure 1**), in combination with loss of EpCAM and retention of E-cadherin indicates *partial EMT* (pEMT) typical of aggressive tumor cells (7). This profile was retained when MPE tumor cells were cultured in the presence of autologous cell free fluid (CFF) or medium supplemented with IL-6 plus IL-8 or TGFβ (**Figure 5C**). Further, addition of the IL-6Rα antagonist tocilizumab to cultures containing CFF completely abrogated tumor EMT, driving tumor cells to transition to epithelioid morphology (**Figures 5C, D**) and indicating that pleural tumor cells have the plasticity to revert from mesenchymal to epithelial states (MET).

### PIT effector activity is unaffected by IL-6Rα antagonism

Pleural T cells, activated and expanded in the presence of CD3/CD28 beads plus IL-2 are potent cytotoxic effectors against autologous tumor as measured by LDH release (**Figure 4**). Addition of graded doses of tocilizumab during PIT activation and expansion did not alter cytotoxic activity (**Figure 5E**). The same PIT cells that were tested for cytolytic activity (**Figure 5E**) were also evaluated for cytokine production by flow cytometry (pooled wells with tocilizumab at 3 and 6 μg/mL) and compared to cytokine production in the absence of tocilizumab (**Figure 5F**). In the presence or absence of tocilizumab, a small proportion of activated CD4 and CD8 pleural T cells produced IL-2 and the majority actively produced IL-10, IFNγ and TNFα (**Figure 5F**), supporting the interpretation that inhibition of the IL-6/sIL-6Rα axis does not hamper immune effector responses.

### Effusions as a source of anti-tumor effectors for adoptive cellular therapy

**Figure 6:**
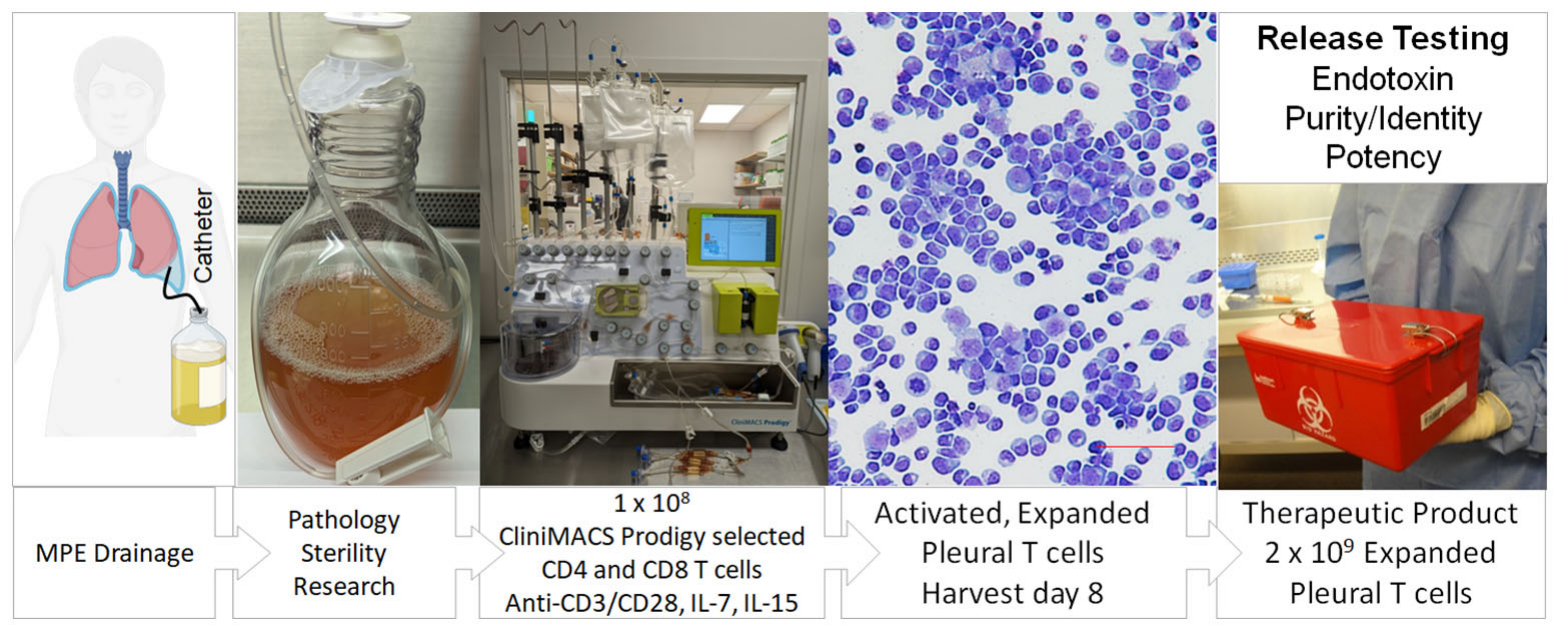
Schema for production of a cellular therapeutic from pleural infiltrating T cells. Patients routinely drain effusions into a sterile vessel using a Pleurex catheter. T cells in the pleural fluid are quantified by flow cytometry and loaded onto the Miltenyi CliniMACS Prodigy, a closed system instrument capable of cell selection and expansion. CD4+ and CD8+ cells are purified by immunomagnetic selection; other cells, including tumor cells are excluded. A volume of selected cells containing 1 x 10^8^ T cells are transferred to a temperature and CO_2_ controlled chamber where they are activated with CD3/CD28 beads and cultured in the presence of IL-7 and IL-15. Eight days later, activated, expanded pleural T cells, which have undergone a 20 to 50-fold expansion, are harvested. In future clinical trials, cells will be tested for endotoxin, activated T cell content (identity), absence of tumor cells (purity) and cytokine secretion (potency) prior to release as a cellular therapeutic.

Adoptive cellular therapy using autologous tumor infiltrating lymphocytes (TIL) has been reported to induce salvage responses in a variety of refractory solid tumors (18). Conventionally, TIL therapy requires large-scale expansion of a small number of T cells grown out from tumor tissue fragments stimulated with high dose IL-2 and anti-CD3 antibody. Using the Miltenyi CliniMACS Prodigy system, a GMP-compliant platform capable of cell selection and expansion in a closed system, we selected 1 x 10^8^ CD4+ and CD8+ cells from a single NSCLC pleural effusion. After immunomagnetic selection the cells were activated with CD3/CD28 beads in the presence of IL-7 and IL-15 (**Figure 6**). Eight days later, activated PIT, which were expanded 30-fold (3 x 10^9^ cells), were harvested and tested for cytotoxicity against autologous tumor targets (cytolytic index of 4.5; **Table 1**). Taken together cGMP expanded PIT exhibited cytolytic ability similar to the series of small-scale *in vitro* culture expansions in the three types of metastatic cancers tested (breast, NSCLC and RCC), supporting the feasibility of creating a cellular therapeutic by short-term expansion of pleural T cells.

## Discussion

The presence of a robust immune infiltrate accompanying virtually all malignant pleural effusions poses a conundrum: why, in the presence abundant T cells and macrophages, does the tumor thrive and attain an aggressive and therapy resistant state (19, 20)? Although MPE are uniformly fatal, high numbers of T cells, and low numbers of neutrophils have been associated with marginally longer survival times (21). The present study reveals that MPE infiltrating T cells are maintained in a quiescent state by the unique pleural immune environment (22), as evidenced by low expression of ICM (**Figures 1**, **3**), scant cytoplasm (**Figure 1**), the 2n DNA content and the predominant effector-memory phenotype (**Figure 2**; **Supplementary Figure 4**). This contrasts with the observation that ICM are expressed on T cells across a wide variety of cancers. A meta-analysis of expression of the ICM PD-1, CTLA4, TIM-3 and LAG-3 revealed that paradoxically, high expression of all markers except CTLA-4 correlated with a modest but consistent survival advantage (23).

Although high ICM expression renders T-cells susceptible to inactivation or apoptotic death, and coexpression of multiple checkpoints are an indicator of exhaustion (24), we found that multiple ICM expression also accompanies generation of effector function, as illustrated by ICM upregulation when PIT are activated *in vitro* in the present series (**Figure 1**). Upregulation of the PD-Ls on activated CD4+ T cells in our series is consistent with the results of Mazerolles et al., who reported that PD-L1 expression on activated CD4 effector T cells is correlated with T-cell proliferation (25), and Trinchieri who observed that immunosuppressive mechanisms typical of the tumor microenvironment are observed in infected tissues during resolution of the immune response to infections (26). The observed low expression of ICM on freshly isolated pleural T cells may explain why immune checkpoint blockers are ineffective in the setting of MPE but may be effective if an activated T-cell therapeutic is contemplated (27).

In this series, comparing the secretomes of expanded PIT with that of cultured tumor and MPE CFF, we can infer that activation with CD3/CD28 plus IL-2 drives a variety of myeloid chemoattractants, TNFα (which together with IFNγ drives M1 polarization), matrix metalloproteases, effector cytokines, and a host of T-cell growth and differentiation factors.

**Figure 7:**
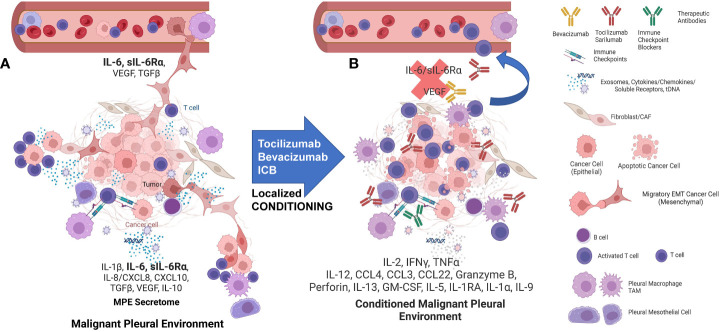
The interactions of MPE tumor, TAM, and T-cells are highly dependent on the local environment but can be conditioned to support anti-tumor immunity and drive effector-memory T cell generation. **(A)** MPE Tumor effects on TAM: Tumor, particularly tumor that has undergone EMT, amplifies and maintains TAM M2-like wound healing polarization. TAM are recruited with tumor-secreted G-CSF, IL-6, IL-8, CCL2/MCP1, CXCL1/GRO, G-CSF, GM-CSF, TGFβ and CD90/CD11b interactions. IL-6 secreted by the tumor and the M2 TAM also acts as a potent growth factor on pleural mesothelium in the presence of soluble IL-6Rα. MPE Tumor and TAM polarization effects on pleural T cells: M2 TAM polarization favors T-cell suppression through cytokines (IL-6, TGFβ, IL-1RA, IL-10) and programmed death ligand-induced apoptosis. The pleural space is isolated from the systemic circulation, permitting the maintenance of very high local cytokine and chemokine levels, creating a pleural immune environment in which potential effector cells are maintained in a quiescent state and tumor is maintained in an aggressive mesenchymal state. EMT tumor-TAM clusters invade lymphatics and vessels, spread and seed distant metastases. **(B)** The pleural environment is conditioned by local delivery of cytokines, activation signals, antibody-based therapeutics and ex-vivo activated PIT, tipping the balance in favor of the immune effector response. Local “conditioning” therapy results in M1 TAM polarization, providing presentation of tumor associated-antigens and costimulatory signals, culminating in the maintenance and expansion of tumor-specific effector T cells. Once the immune suppression has been lifted, antigen presenting cells and effector-memory T cells can traffic to the draining lymph nodes where responding T cells can proliferate and differentiate. Once this has occurred, tumor specific effector T cells can be expected to home to other sites of metastasis. Figure was generated in BioRender.

On the negative side, expanded PIT also secrete cytokines that can promote tumor EMT, including sIL-6Rα, IL-8 and IL-6. IL-6 and its secreted receptor sIL-6Rα are increasingly recognized as master cytokines (28, 29), upstream of a wide array of inflammatory processes, including pathologies as diverse as cytokine release syndrome (30), SARS-CoV2 acute respiratory distress (31), acute allograft rejection (32), rheumatoid arthritis (33), asbestosis (34) and cachexia (35). Complexes of soluble IL-6/IL-6Rα elicit responses from gp130-expressing cells that lack the complete IL-6 receptor (known as trans-signaling) (28, 36). Our secretomic data supports the role of IL-6/sIL-6Rα receptor trans-signaling as the key driver of tumor EMT and associated therapy resistance and increased metastatic potential (**Figure 7**). Our current findings confirm our previous findings in NSCLC-associated MPE (4) and reveal a profound degree of cytokine-chemokine polarization dominated by IL-6 and sIL6Rα at near nM concentrations (**Figures 2**, **5A**). Neutralizing pleural IL-6 or IL-6Rα activity with therapeutic antibodies may not only diminish IL-6-driven aggressive tumor behavior associated with EMT (37) (**Figures 5B–D**), but may also reverse downstream negative regulation of tumor-specific immune effector responses (**Figures 5E, F**), thereby enhancing the efficacy of other immune oncology therapies (38). Although long-term antagonism of IL-6Rα is immunosuppressive (39), single dose exposure has been shown to break the cytokine storm associated with CAR-T therapy without compromising effector responses (40) or incurring serious adverse effects (41). IL-6R antagonism has also recently been shown to act synergistically with ICM blockers in cancer therapy (38). Taken together, localized intra-pleural administration of anti-IL-6 or anti-IL-6Rα may likewise be expected to exert profound effects on the malignant pleural environment (22, 38).

In our database of MPE secretomics from a variety of cancers (n = 396), we have observed few statistically significant differences between cancers, emphasizing the concept that the pathobiology of MPE is driven by the intrapleural milieu, rather than by the biology of the individual cancer type. Cultured tumor cells appear to mirror many of the cytokines and chemokines found in MPE. Like Expanded PIT, they produced high levels of IL-8, granzyme B, IL-6 and MMP7. Tumor cells also secrete high levels of B7-H6, E-cadherin and eotaxin. The proinflammatory protease MMP7 is associated with breakdown of the extracellular matrix, wound healing and tumor metastasis (42). Although granzyme B is known chiefly as an effector molecule which, in conjunction with perforin, mediates T-cell and NK-cell cytotoxic effector responses, soluble granzyme B is also associated with the immunosuppressive tumor niche (43) and is secreted directly by breast (44) and urothelial tumor cells, where it’s presence is associated with tumor EMT and invasion (45, 46). B7-H6 is associated with tumor progression and metastasis in ovarian (47) and breast (48) cancer, but when expressed by tumor, also may promote recognition by NK cells (49). E-cadherin is important for development of distant metastases and appears to be downregulated and then re-expressed once cancer has spread to distant sites (50, 51). Here we show that freshly isolated MPE tumor cells continue to express E-cadherin even as they transit into a state that has been termed pEMT (7) (**Figure 5B**; **Supplementary Figure 1**). We speculate that sustained IL-6/IL-6Rα signaling during EMT-MET plasticity states further drives the modification of MPE environment (52), amplifying the aggressive MPE tumor state. Although eotaxin (CCL11) is known primarily as a chemotactic factor for eosinophils, high plasma concentrations are associated with metastasis and invasion in breast (53), ovarian (54) and colorectal cancers (55), again significantly contributing to generation of a maladaptive pleural environment. Taken together, our results indicate that low passage cultured tumor cells continue to secrete cytokines and chemokines that promote EMT and maintain T-cell quiescence. This potent immunosuppressive effect appears to be fundamentally different from ICM-mediated exhaustion (**Figure 1**). Although Regulatory T cells (T-reg) have been reported in MPE (56), they were below the limit of GeoMx detection (**Figure 3**). This is consistent with our own flow cytometric data in which CD25+ cells constitute 0.75 ± 0.39% (Mean ± SEM, N = 11) of CD4+ T cells (**Supplementary Table 4**). Functional T-regs are a subset of CD4+/CD25+ T cells and would represent an even smaller fraction of CD4+ T cells. The paucity of T-reg, in an environment rich in CCL22 (**Figure 2**) may be explained by the absence of CCL4 expression on pleural CD4+ T cells (**Supplementary Table 4**). In breast cancer, CCL22 has been shown to be chemoattractive for CCR4+, but not CCR4- T-reg (57).

Our published findings in NSCLC, mesothelioma (4) and ovarian (22) MPE reveal a profound degree of polarization dominated by near nM concentrations of IL-6/sIL6Rα, IL-8, IL10, VEGF, FGF2 and CXCL10. We have previously shown that IL-6 is produced by the tumor, as well as by pleural macrophages (37). Thus, the same T-cell, macrophage and epithelial-cell mediated secretome that normally promotes tissue maintenance and wound healing in normal physiology, becomes self-sustaining and maladaptive when neoplastic cells contribute to the process.

From a clinical perspective, the most salient finding of this study is that, when removed from the immunosuppressive environment of the pleural cavity, PIT can be activated and expanded *in vitro* using conventional methods. In agreement with our colleagues at the University of Pittsburgh (58), we have shown that after *in vitro* activation, MPE cells display potent cytolytic activity for autologous tumor, indicating their promise as a cellular therapeutic. Activation to cytolytic T cells was observed after as little as 2 days in culture (**Table 1**), too short an interval to induce substantial expansion. The fact that stimulated CD4+ pleural T cells dimly coexpressed CD8, perforin and granzyme B (**Supplementary Figure 2**) raises the possibility that these cells can recognize antigen in the context of MHC class I (59), a property usually associated with CD8+ effector cells. The potency of cytotoxic T cells can be quantified by their cytolytic index, which accounts for both the percentage of target cells killed and the ratio of effector to target cells required. By this metric, killing was impressive (cytolytic index range = 4.2 – 78.5). To put these values into context, in the original report of this metric, indices ranged from 2.8 to 48 in mice immunized with an allogeneic cell line (10). Taken together, the demonstration of perforin and granzyme B in PIT (**Supplementary Figure 2**), a predominant effector-memory differentiation state (**Supplementary Figure 4**), and the ease with which cytotoxic effector function and effector cytokines are generated *in vitro* (**Figure 5F**), indicates that the immediate precursors of anti-tumor effector T cells are numerous but suppressed in MPE.

Because virtually all MPE are accompanied by a mononuclear immune infiltrate, they are uniquely suited to the generation of a cellular therapeutic (**Figure 6**). Conventionally, their manufacture requires large-scale expansion of a small number of T cells grown out from tumor tissue fragments. Activated T cells emerge from these tissues and are expanded for 3-5 weeks, until they reach the density required to seed a larger vessel (5-10 x 10^6^ cells total). Rapid expansion typically requires an additional 14 days in culture, for a total expansion time of 5 to 7 weeks. In contrast, therapeutic drainage of MPE frequently yields on the order of 0.25 to 0.5 x 10^6^ pleural T cells/mL. In our experience, yields of 1-5 x 10^8^ pleural T cells are common, especially for the first drainage. Thus, prior to *in vitro* expansion, a single drainage can approach 10-50% of therapeutic doses of pleural T cells. Here we show a *proof-of-concept* short-term *ex vivo* expansion in an automated GMP-compatible device, the ClinMACS Prodigy (**Figure 6**), demonstrating that a single drainage is sufficient to obtain therapeutic doses of pleural T cells following short-term activation and expansion. Reduced culture time (8 days) may better preserve T-cell diversity, cytokine polyfunctionality and cytolytic effector function (**Figures 5E, F**). In future clinical trials, the *ex vivo* short-term expanded pleural T cells will be tested for endotoxin, activated T-cell content (identity), absence of tumor cells (purity) and cytokine secretion (potency) prior to release as a cellular therapeutic (21 CFR Chapter I Subchapter F, Part 610 (2023)). We anticipate that the *ex vivo* activated cells would best be re-instilled into the pleural cavity after local conditioning to neutralize the IL-6/IL-6Rα axis (22). Potential advantages of intrapleural administration of activated pleural T cells over conventionally expanded TIL include greatly simplified and rapid manufacture, and ability to monitor effector function in subsequent MPE drainages. Although our small-scale expansions tested several activation strategies, we chose IL-7 plus IL-15 for our clinical scale expansion as it may favor differentiation to a T-cell memory phenotype (60) and eliminate the need for IL-2 administration to promote TIL expansion *in vivo* (61, 62).

**Figure 7A** illustrates our emerging understanding of the immunopathology of malignant effusions. MPE occur in a contained pleural immune environment that functions as a bioreactor into which potential effector cells are recruited but potently suppressed and in which the tumor is maintained in an aggressive mesenchymal state. Once initiated by tumor, interactions between tumor, TAM, mesothelial cells, and T cells amplify and sustain this maladaptive local environment. EMT tumor/tumor associated macrophage (TAM) clusters invade lymphatics and vessels to disseminate and seed distant metastases. As we have previously observed (22), even the most effective cellular therapeutic will have reduced efficacy if delivered to a highly immunosuppressive environment, such as the pleural cavity. Local delivery of some combination of cytokines, activation signals, antibody-based therapeutics, localized mRNA therapeutics, or *ex-vivo* activated PIT, may break this positive feedback loop, tipping the balance in favor of the immune system (**Figure 7B**). Such local “conditioning” may also repolarize TAM to an M1 state, promoting presentation of tumor associated-antigens, providing costimulatory signals, and culminating in expansion of tumor-specific effector T cells. We envision that once the immune suppression has been breached, antigen presenting cells and effector-memory T cells can traffic to the draining lymph nodes where responding T cells can proliferate, differentiate, and home to other metastatic sites. We expect that a successful approach to immunotherapy of MPE will require combining a cellular therapeutic with pleural conditioning agents such as immune checkpoint blockers and cytokine antagonists. Our data support IL-6 as an upstream master cytokine in the immunopathology of MPE, and the IL-6/IL-6R axis (22) as an attractive therapeutic target.

## Data availability statement

The raw data supporting the conclusions of this article will be made available by the authors, without undue reservation.

## Ethics statement

The studies involving human participants were reviewed and approved by University of Pittsburgh IRB. The patients/participants provided their written informed consent to participate in this study.

## Author contributions

AD and VD designed the experiments, analyzed the data, created the figures and wrote the manuscript. JDL, IS, SG and DB contributed their clinical expertise and facilitated collection of the clinical samples used in this study. JL contributed expertise on large-scale T-cell expansion and donated the CliniMACS Prodigy reagents and the instrumentation time. All authors contributed to the article and approved the submitted version.
