# Supplementary material for: A maladaptive pleural environment suppresses preexisting anti-tumor activity of pleural infiltrating T cells
Source: Front Immunol. 2023 Mar 30;14:1157697. doi: 10.3389/fimmu.2023.1157697 (PMC10097923; doi:10.3389/fimmu.2023.1157697)
Supplement: Supplementary file 1 [file DataSheet_1.pdf]

A Maladaptive Pleural Environment Suppresses Preexisting Anti-Tumor Activity of Pleural Infiltrating T cells. Donnenberg et al. 2023.

Supplementary Table 1. Patient Demographics. Sample IDs correspond to those listed in the manuscript.

Sample ID	Diagnosis	Stage	Age	Sex	Treatment Resistant (Effusion)*	Figure/Table
NPF28	Mitral Valve replacement	NA	74	M	NA	F1
NPF29	Aortic Valve replacement	NA	52	M	NA	F1
NPF30	Aortic Aneurism	NA	53	M	NA	F1
MPE29	Non-Small Cell Lung Cancer	IV	89	F	Yes	ST4
MPE66	Non-Small Cell Lung Cancer	IV	46	F	Yes	ST4
MPE67	Non-Small Cell Lung Cancer	IV	66	F	No prior treatment, Naïve	ST4
MPE83	Breast Cancer (ER-PR-Her2-)	IV	69	F	Yes	ST4
MPE094	Breast Cancer (ER-PR-Her2-)	IV	59	F	Yes	F3
MPE104	Non-Small Cell Lung Cancer	IV	67	F	Yes	ST4
MPE107	Breast Cancer (ER+PR-Her2+)	IV	82	F	Yes	ST4
MPE108	Non-Small Cell Lung Cancer, primary with MPE	IIIA, IV	83	M	No prior treatment, Naïve	ST4
MPE111	Breast Cancer, poorly differentiated (ER-PR unknown Her2-)	IV	59	F	Yes	ST4
MPE128	Breast Cancer, metastatic to the lung and the pleura	IV	59	F	No prior treatment, Naïve	ST4
MPE136	Non-Small Cell Lung Cancer, primary with MPE	IV	63	M	No prior treatment, Naïve	ST4
MPE138	Breast Cancer, poorly differentiated (ER-PR unknown Her2-)	IV	59	F	Yes	ST4
MPE157	Breast Cancer (ER+PR+Her2-)	IV	44	F	Yes	F3
MPE163	Non-Small Cell Lung Cancer	IV	58	M	Yes	F1
MPE169	Breast Cancer (ER-PR-Her2-)	IV	62	F	Yes	F3
MPE175	Breast Cancer (ER+PR+Her2unknown)	IV	67	F	Yes	F3
MPE176	Cholangial Carcinoma	IV	53	M	Yes	F1

MPE177	Non-Small Cell Lung Cancer	IV	63	M	Yes	F1
MPE178	Esophageal Cancer	IV	68	M	No prior treatment, Naïve	F1
MPE179	Breast Cancer (ER+PR unknown, HER2-)	IV	67	F	Yes	F1
MPE185	Breast Cancer	IV	NA	F	Yes	F1
MPE192	Breast Cancer, Invasive Luminal Carcinoma (ER+PR+ Her2-)	IV	71	F	Yes	F3
MPE195	Breast Cancer (ER+PR+Her2-)	IV	66	F	Yes	F1
MPE197	Breast Cancer (ER-PR-Her2-)	IV	65	F	Yes	F1
MPE199	Breast Cancer (Fluid positive for malignant cells)	IV	69	F	Yes	F1
MPE200	Breast Cancer (ER+PR+)	IV	76	F	Yes	F1, F3
MPE201	Breast Cancer (ER-PR-Her2-)	IV	55	F	Yes	F3
MPE202	Breast Cancer (ER+PR+)	IV	NA	F	Yes	F3
MPE205	Breast Cancer (ER+PR+Her2+)	IV	54	F	Yes	F1, F3
PE207	Mitral valve operation	NA	53	F	NA	F3
MPE208	Breast Cancer (ER+PR-)	IV	94	F	No prior treatment, Naïve	F1, F2, SF4, SF5
MPE211	Renal Cell Carcinoma	IV	70	F	Yes	F1, F2, SF4, SF5
MPE212	Breast Cancer (ER+PR+Her2-)	IV	71	F	Yes	F1, F2, F 4, F5, SF1, SF2, SF5
MPE213	Non-Small Cell Lung Cancer	IV	90	M	Yes	F1
MPE215	Non-Small Cell Lung Cancer	IV	74	F	No prior treatment, Naïve	F6, SF3
***Treatment Resistant/Naive applies only to the malignant effusion episode**						

Supplementary Table 2. Antibodies used for flow cytometry studies. T-Cell_IC_CTX = reagents used for immune checkpoint and studies on cytotoxic effector cells. Tumor_EMT = reagents used for assessment of epithelial to mesenchymal transition on cultured tumor cells.

Marker	Fluorochrome	Catalog #	Vendor	T-Cell_IC_CTX	Tumor_EMT
CD27	APC	581786	BD Pharmingen	x	
CD19	A700	A78837	Beckman Coulter	x	
CD326	APC Cy7	324234	BioLegend		x
ipH3	Alexa Fluor 647	558609	BD Pharmingen		x
CD279	PE	557946	BD Pharmingen	x	
CD274	PE	557924	BD Pharmingen	x	
CD273	PE	558066	BD Pharmingen	x	
LAG3	PE	585616	BD Pharmingen	x	
TIGIT	PE	372703	BioLegend	x	
TIM3	PE	566346	BD Pharmingen	x	
CTLA4	PE	2282	Immunotech	x	
CD137	PE	555956	BD Pharmingen	x	
CD4	PE	IM0449U	Beckman Coulter	x	
iVimentin	PE	ab49918-100	abcam		x
CD8	ECD	660478	Beckman Coulter	x	
E-CAD	PE Dazzle 594	147315	BioLegend		x
CD56	PE Cy5	IM2654U	Beckman Coulter	x	
CD44	PE Cy7	ab46793	abcam		x
CD14	PerCP Cy5.5	325622	BioLegend	x	
iCTK	FITC	IM2356U	Beckman Coulter	x	x
DNA	DAPI	D1306	Invitrogen	x	x
CD90	BV650	740585	BD OptlBuild		x
CD3	BV711	563725	BD Horizon	x	
CD4	BV785	317442	BioLegend	x	
CD45	BUV395	563792	BD Horizon	x	x
CD45RA	BUV737	612846	BD Horizon	x	
iPerforin	APC Cy7	308128	BioLegend	x	
iGranzyme B	PE Cy7	372214	BioLegend	x	

Supplementary Table 3. Comparison of immune checkpoint molecule (ICM) expression between groups. The data are from Figure 1A. Between group differences (percent positive) and p-values are shown. Analysis of variance was performed assuming equal variances. Tukey's honestly significant difference test (Tukey's HSD) was used to test differences among sample means. These results were then Bonferroni corrected for multiple comparisons (8 different ICMs). Significant differences are highlighted.

		PD-1		PDL-1		PDL-2		LAG3		TIM3		CTLA4		TIGIT		41BB	
Comparison		Difference	p-value	Difference	p-value	Difference	p-value	Difference	p-value	Difference	p-value	Difference	p-value	Difference	p-value	Difference	p-value
CD4 NPF	CD4 MPE	-16.0	1.0000	-5.0	1.0000	0.3	1.0000	-0.8	1.0000	0.5	1.0000	-3.6	1.0000	5.5	1.0000	-1.2	1.0000
CD4 NPF	CD4 In Vitro Activated	-52.1	0.0566	-58.8	0.0133	-30.3	0.1960	-64.1	0.0109	-23.3	1.0000	-42.4	0.0747	-61.4	0.0015	-24.2	1.0000
CD4 NPF	CD8 NPF	-2.0	1.0000	0.1	1.0000	0.0	1.0000	0.0	1.0000	0.1	1.0000	0.1	1.0000	-5.3	1.0000	0.0	1.0000
CD4 NPF	CD8 MPE	-15.5	1.0000	-1.3	1.0000	0.3	1.0000	-0.7	1.0000	0.7	1.0000	-1.3	1.0000	5.6	1.0000	-1.0	1.0000
CD4 NPF	CD8 In Vitro Activated	-26.3	1.0000	-21.7	1.0000	-5.2	1.0000	-73.6	0.0018	-26.6	1.0000	-14.1	1.0000	-71.5	0.0002	-21.1	1.0000
CD4 MPE	CD4 In Vitro Activated	-36.1	0.0071	-53.9	0.0040	-30.7	0.0291	-63.3	0.0010	-23.8	1.0000	-38.8	0.0038	-67.0	0.0001	-23.0	0.9765
CD4 MPE	CD8 NPF	14.0	1.0000	5.1	1.0000	-0.3	1.0000	0.8	1.0000	-0.4	1.0000	3.7	1.0000	-10.8	1.0000	1.2	1.0000
CD4 MPE	CD8 MPE	0.5	1.0000	3.6	1.0000	0.0	1.0000	0.2	1.0000	0.2	1.0000	2.3	1.0000	0.0	1.0000	0.2	1.0000
CD4 MPE	CD8 In Vitro Activated	-10.3	1.0000	-16.7	1.0000	-5.6	1.0000	-72.7	0.0001	-27.1	0.5571	-10.5	1.0000	-77.0	0.0000	-19.9	1.0000
CD4 In Vitro Activated	CD8 NPF	50.1	0.0854	58.9	0.0131	30.4	0.1951	64.1	0.0110	23.4	1.0000	42.5	0.0736	56.2	0.0048	24.2	1.0000
CD4 In Vitro Activated	CD8 MPE	36.6	0.0058	57.5	0.0016	30.7	0.0291	63.5	0.0010	24.0	1.0000	41.2	0.0016	67.0	0.0001	23.2	0.9350
CD4 In Vitro Activated	CD8 In Vitro Activated	25.8	0.1562	37.2	0.0302	25.1	0.0197	-9.4	1.0000	-3.3	1.0000	28.3	0.0619	-10.0	1.0000	3.1	1.0000
CD8 NPF	CD8 MPE	-13.5	1.0000	-1.4	1.0000	0.3	1.0000	-0.6	1.0000	0.6	1.0000	-1.3	1.0000	10.8	1.0000	-1.0	1.0000
CD8 NPF	CD8 In Vitro Activated	-24.3	1.0000	-21.8	1.0000	-5.2	1.0000	-73.5	0.0018	-26.7	1.0000	-14.2	1.0000	-66.2	0.0005	-21.1	1.0000
CD8 MPE	CD8 In Vitro Activated	-10.8	1.0000	-20.3	1.0000	-5.6	1.0000	-72.9	0.0001	-27.3	0.5306	-12.9	1.0000	-77.0	0.0000	-20.1	1.0000

Supplementary Figure 1. Passage 0 MPE tumor cells used for targets in the cytotoxicity assay. MPE cells were suspended in MEGM medium and plated in a T75 flask. Non-adherent cells were harvested 24h later for PIT activation, after which tumor cells were refluided with MEGM containing 10% human AB serum and antibiotics. The photomicrographs were taken on day 9 prior to tumor cell harvest. Contrast, brightness and sharpness were enhanced using Photoshop. Tumor cells were gently trypsinized (0.25% trypsin, 2.2 mM EDTA), counted and plated in 96-well plates for cytotoxicity; the remainder was stained for flow cytometry (main article Figure 5B).

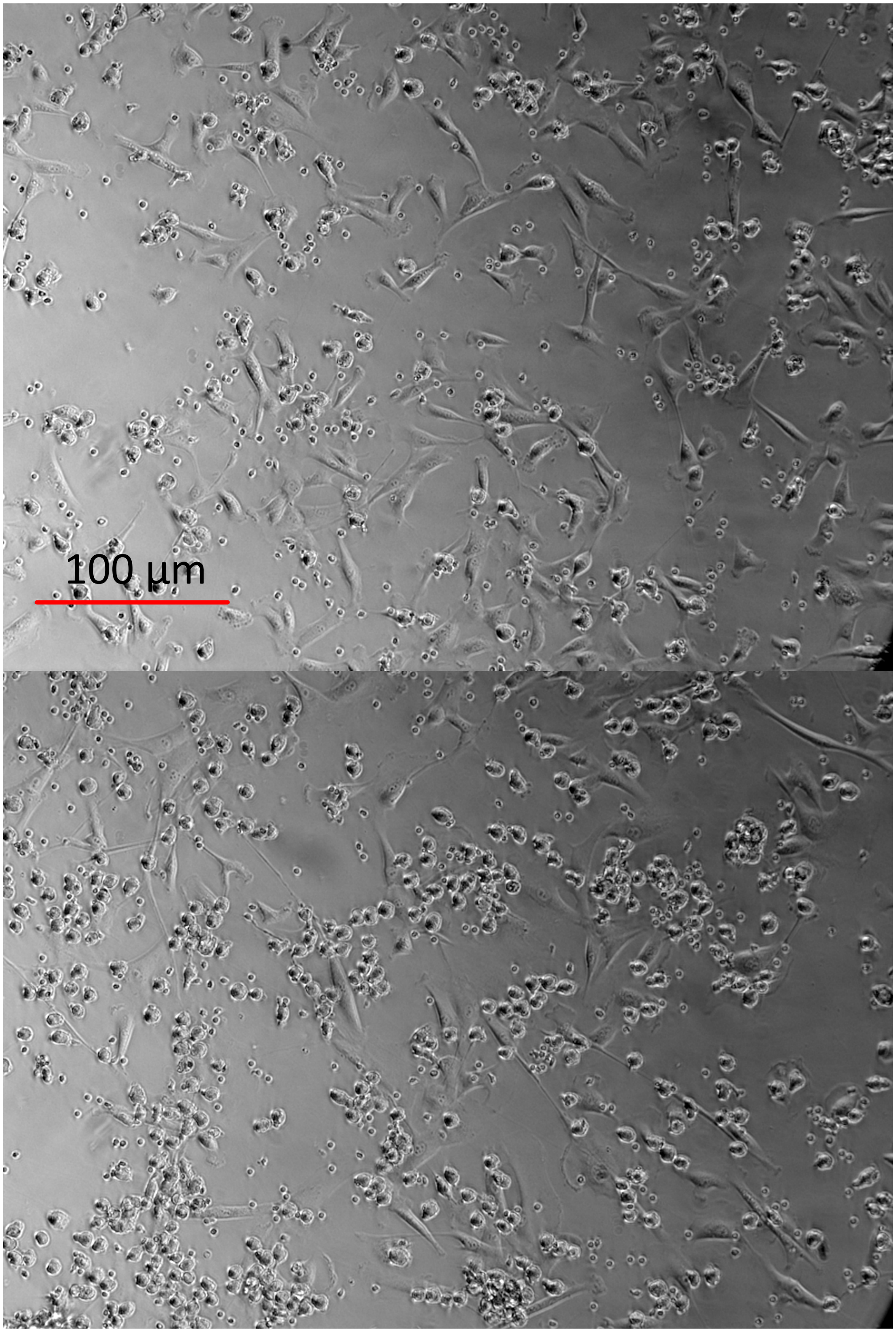

Supplementary Figure 2. Characterization of residual tumor cells and cytotoxic effectors after 4h of coincubation. Non-adherent cells from wells at E:T 50, 25, and 12.5:1 and E:T 6:1 were aspirated, pooled and stained for flow cytometry. (A) Diagram of the 96-well plate. Photomicrographs (enhanced contrast, brightness, sharpness) show representative wells after aspiration and document tumor killing at E:T as low as 3:1. Well B11 shows pleural T cells before aspiration. (B) Flow cytometry on pooled non-adherent cells. Wells from rows A and B represent stimulated, culture expanded pleural T cells (PIT). Wells from rows C and D represent PIT that were held in culture without stimulation. Stimulated cells at high E:T (A 1-9) had dim expression of CD8 on CD4+ T cells and high coexpression of granzyme B and perforin on CD4+ and CD8+ T cells. CD4+ T cells had high expression of PD-1 (CD279) compared to CD8. Despite PD-1 expression, stimulated PIT were potent cytotoxic effectors. Stimulated cells plated in the absence of tumor (B 10-12) had similar profiles, albeit with lower coexpression of granzyme B and perforin. Unstimulated cells (rows C and D) lacked CD4+ CD8dim cells, did not express granzyme B or perforin, but upregulated PD-1 as a consequence of culture.

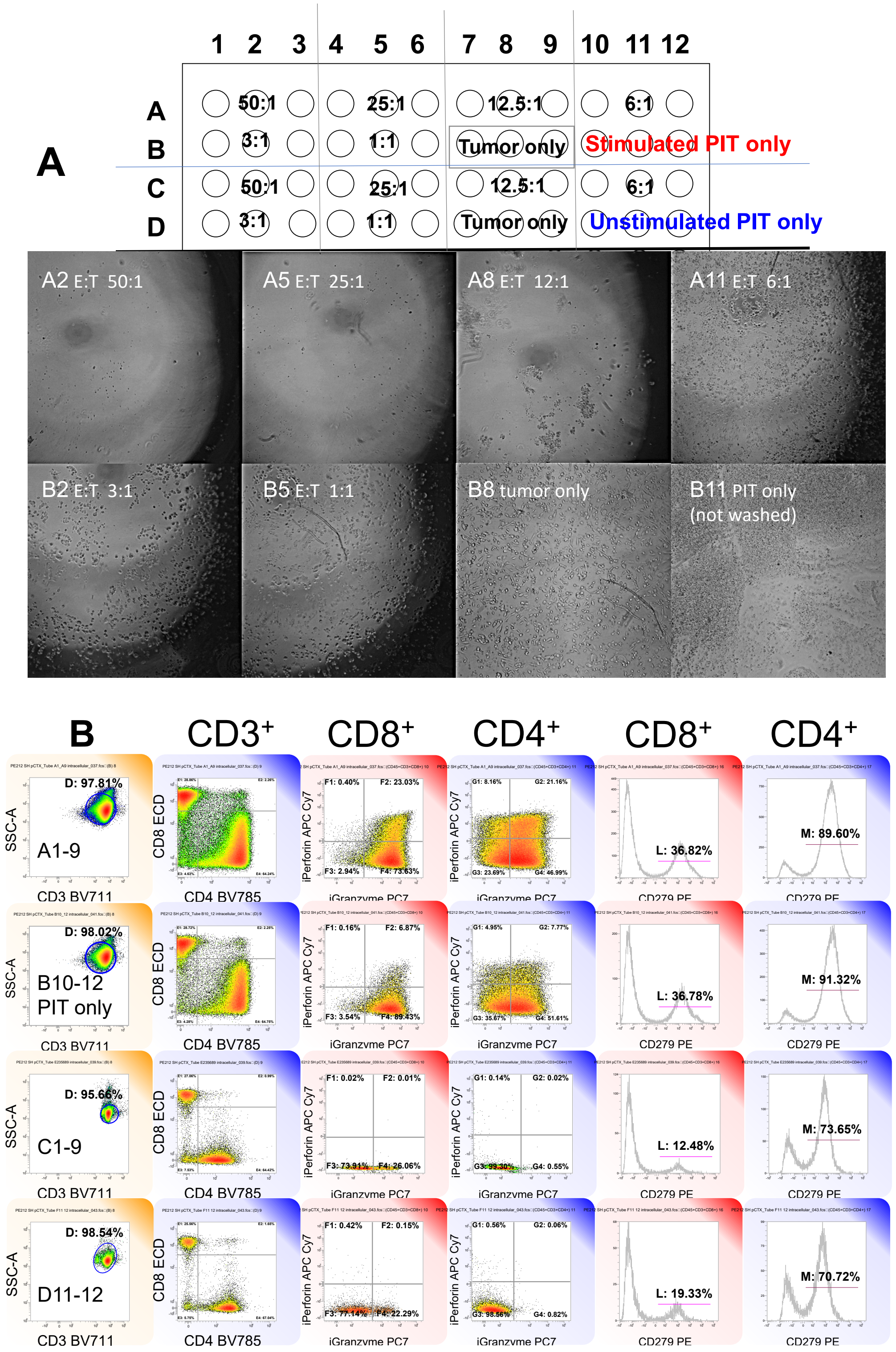

Supplementary Figure 3. Nonadherent pleural cells contain pleural lymphocytes and macrophages. Nonadherent cells were harvested after 24h culture of MPE cells (non-small cell lung cancer) in BEGM medium. Non-adherent cells were activated with anti-CD3/CD28 beads (small red spheres) in the presence of IL-7 and IL-15 in RPMI-1640 medium supplemented with 10% human AB serum. Left panel (4 days of activation culture); Right panel (8 days of activation culture). A sample of morphologically identifiable macrophages are indicated by arrows. Many have phagocytosed the beads.

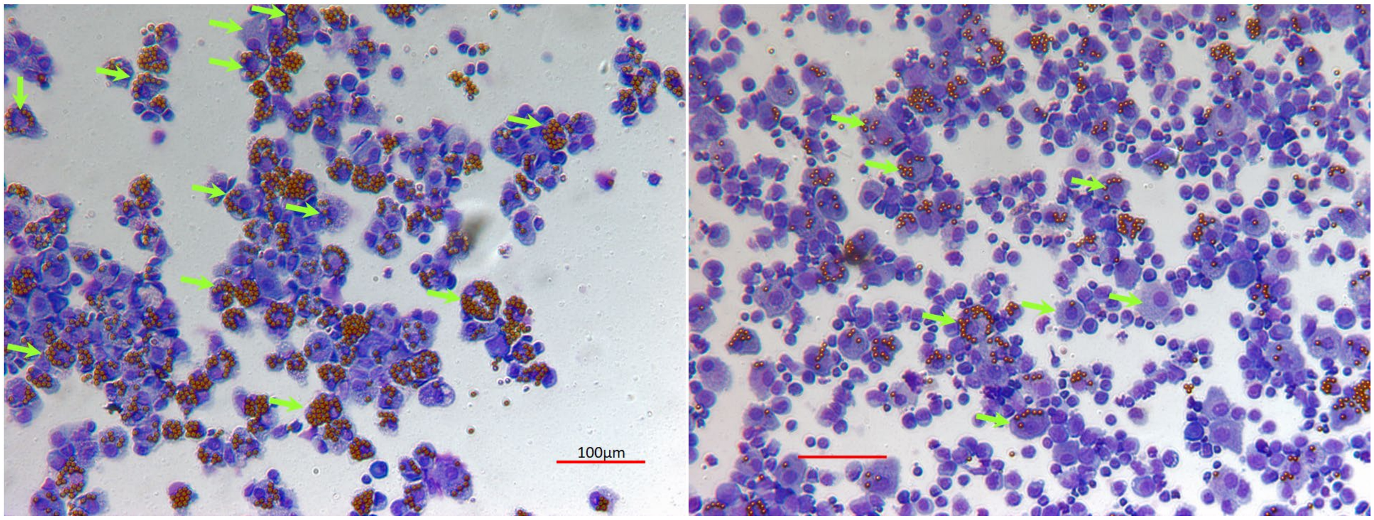

Supplementary Figure 4. Freshly isolated pleural T-cells are effector memory phenotype and generate effector cells after *in vitro* expansion. Columns from left to right: Gating strategy; Fresh pleural effusion cells from a therapy naïve patient with ER+PR- Breast Cancer CD8 and CD4+ T cells are predominantly CD45RA-/CD27- effector-memory cells; After activation and expansion for 14 days a distinct population of effector cells (CD45RA+/CD27-) emerges; After 4h exposure to tumor in the cytotoxicity assay (E:T = 10:1, main manuscript Table 1), CD4 and CD8 effector cells are reduced in number, but still present.

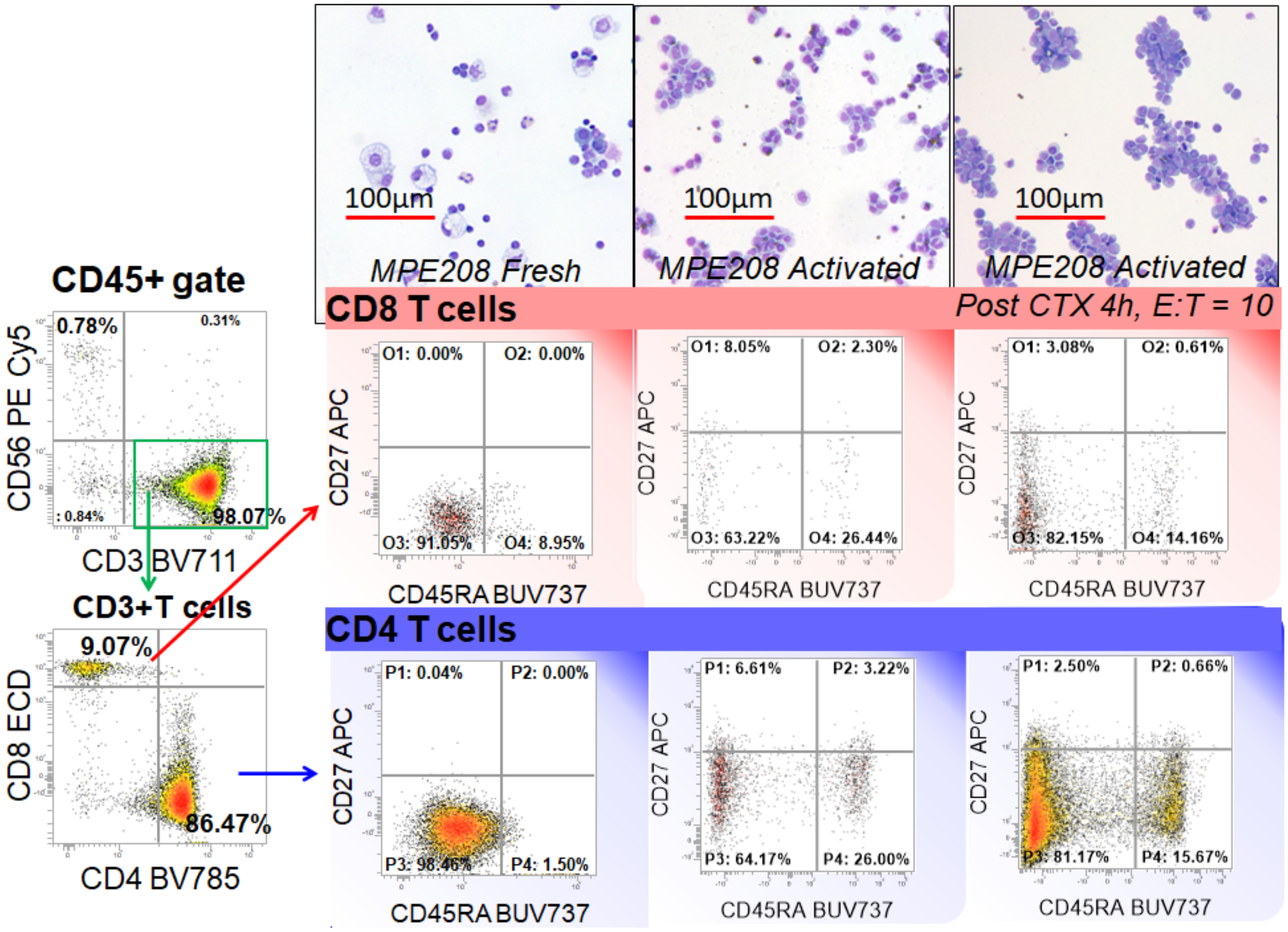

Supplementary Figure 5. Secretome of culture supernatants of in vitro activated PIT (n=10), primary MPE tumor cell cultures (n=39) and MPE cell-free fluid (n=396). Data within heatmap boxes are expressed as $\log_{10}$ pg/mL mean concentrations. Data are ordered according to pM concentration in activated PIT as expressed in the main manuscript (Figure 2). Cytokine and chemokine molecular masses (kD), determined from the literature (Source) do not account for isoforms, glycosylation or other sources of variability.

Analyte	Activated PIT	Cultured Tumor	MPE	MW (kD)	Source
CXCL8/IL8	4.2	4.3	2.6	8.9	https://www.peprotech.com/en/recombinant-human-il-8-77-aa-cxcl8
CCL4/MIP-1β	4.1	0.7	1.4	7.6	https://www.peprotech.com/en/recombinant-human-mip-1-ccl4
CXCL10/IP10	4.1	2.6	3.8	8.6	https://www.peprotech.com/en/recombinant-human-ip-10-cxcl10
CCL3/MIP-1α	4.1	1.9	1.4	7.8	https://www.peprotech.com/gb/recombinant-human-mip-1-ccl3
CCL22/MDC	3.9	1.2	2.7	8	https://www.peprotech.com/gb/recombinant-human-mdc-67-aa-ccl22
CCL2/MCP1	3.7	4.3	3.8	8.6	https://www.peprotech.com/gb/recombinant-human-mcp-1-ccl2
CXCL1/GRO	3.5	4.1	2.8	7.8	https://www.peprotech.com/gb/recombinant-human-gro-mgsa
GranzymeB	4.0	1.6	2.7	32	https://en.wikipedia.org/wiki/Granzyme_B
IL13	3.6	-1.5	-0.1	13	https://en.wikipedia.org/wiki/Interleukin_13
CD25	3.8	1.6	3.9	24.8	https://www.peprotech.com/gb/recombinant-human-sil-2-receptor-cho-cell-derived
TNFα	3.6	0.9	1.6	17	https://www.peprotech.com/gb/recombinant-human-tnf-
IL2	3.5	-0.5	0.1	15.5	https://www.peprotech.com/gb/recombinant-human-il-2
IFNγ	3.5	0.2	1.0	16.8	https://www.peprotech.com/gb/recombinant-human-ifn-2-2
GM-CSF	3.4	2.2	1.1	14.6	https://www.peprotech.com/gb/recombinant-human-gm-csf
IL6	3.3	4.1	3.6	20.9	https://www.peprotech.com/gb/recombinant-human-il-6
sIL6Rα	3.6	2.0	4.1	37.9	https://www.peprotech.com/gb/recombinant-human-sil-6-receptor-cho-cell-derived
Perforin	3.7	1.2	3.3	70	https://www.sciencedirect.com/topics/neuroscience/perforin
CD155	3.7	2.8	3.9	70	https://datasheets.scbt.com/sc-27755.pdf
MMP7	3.2	2.4	3.4	30	https://en.wikipedia.org/wiki/MMP7
IL5	3.1	-1.6	1.0	26.5	https://www.peprotech.com/gb/recombinant-human-il-5
MMP9	3.5	1.8	2.1	92	https://www.ncbi.nlm.nih.gov/pmc/articles/PMC4518881/#:~:text=MMP%2D9%20is%20secreted%20as%20an%20inactive%20enzyme%20(proMMP%2D,molecular%20weight%20of%2092%20kDa.
IL10	2.8	0.6	2.3	18.6	https://www.peprotech.com/gb/recombinant-human-il-10
IL1RA	2.7	0.9	1.7	17.2	https://www.sigmaaldrich.com/US/en/product/sigma/srp3084
IL1α	2.6	0.9	1.3	18	https://en.wikipedia.org/wiki/Interleukin_1-alpha
IL9	2.5	-0.7	0.0	14	https://www.peprotech.com/gb/recombinant-human-il-9
CD137	2.7	1.3	2.6	27.9	https://www.sinobiological.com/resource/cd137/proteins
CD134	2.6	0.4	2.9	22	https://www.acrobiosystems.com/P885-Human-OX40--TNFRSF4--CD134-Protein-His-Tag-%28MALS-verified%29.html#:~:text=This%20protein%20carries%20a%20polyhistidine,calculated%20MW%20of%2022.0%20kDa.
MMP3	3.0	1.2	3.8	54	https://en.wikipedia.org/wiki/MMP3
MMP12	3.1	1.1	2.6	80	https://www.abcam.com/recombinant-human-mmp12-protein-ab131994.html
MMP1	2.8	3.1	2.9	54	https://en.wikipedia.org/wiki/Interstitial_collagenase
IL4	2.2	0.5	0.4	15.1	https://www.peprotech.com/gb/recombinant-human-il-4
EGF	1.7	1.7	0.5	6	https://en.wikipedia.org/wiki/Epidermal_growth_factor
IL1β	2.1	1.8	0.9	17.3	https://www.peprotech.com/gb/recombinant-human-il-1-2
TGFβ1	2.2	2.8	3.2	25	https://www.peprotech.com/gb/recombinant-human-tgf-1-hek293-derived
CCL11/EOTAXIN	1.6	1.6	2.0	8.3	https://www.peprotech.com/gb/recombinant-human-eotaxin-3-ccl26
CD276	2.4	2.4	3.0	57.2	https://www.phosphosite.org/proteinAction?id=2612202&showAllSites=true
MMP2	2.5	1.9	3.5	72	https://en.wikipedia.org/wiki/MMP2
MMP10	2.3	1.9	2.1	54	https://www.abcam.com/mmp10-antibody-ab38930.html#:~:text=Predicted%20molecular%20weight%3A%2054%20kDa,1%2F5000%20for%20chemiluminescent%20substrates.
IL17A	2.0	0.7	0.3	31.3	https://www.peprotech.com/gb/recombinant-human-il-17-il-17a
B7-H6	1.9	2.3	2.9	28.6	https://www.acrobiosystems.com/P2598-Human-B7-H6--NCR3LG1-Protein-His-Tag.html
IL3	1.6	-1.0	-0.4	15	https://www.peprotech.com/gb/recombinant-human-il-3
MCP3	1.5	1.3	1.2	13	https://en.wikipedia.org/wiki/CCL7 (4 different glycotypes)
CD40L	1.6	0.9	1.5	16.3	https://www.peprotech.com/gb/recombinant-human-scd40-ligand
TNFβ	1.7	-1.9	1.3	25	https://en.wikipedia.org/wiki/Lymphotoxin_alpha
GCSF	1.5	2.9	2.4	18.7	https://www.peprotech.com/gb/recombinant-human-g-csf
TGFα	0.8	0.7	0.5	5.5	https://www.peprotech.com/gb/recombinant-human-tgf-
ECadherin	2.0	0.3	2.8	97	https://www.abcam.com/e-cadherin-antibody-cdh11525-ab219332.html#:~:text=Predicted%20molecular%20weight%3A%2097%20kDa.
VEGF	1.6	1.6	2.3	38	https://www.peprotech.com/gb/recombinant-human-vegf-b (VEGF-A dimer)
41BBL/TNFSF9	1.1	-0.7	-0.3	19.5	https://www.peprotech.com/gb/recombinant-human-4-1bb-ligand
CX3CL1/FRACTALKINE	0.6	-1.1	1.8	8.5	https://www.peprotech.com/gb/recombinant-human-fractalkine-cx3cl1
IL7	0.9	1.2	1.6	17.5	https://www.peprotech.com/gb/recombinant-human-il-7
CD226	1.4	1.4	1.3	63	https://en.wikipedia.org/wiki/CD226
sCD40L	0.3	-1.3	1.0	16.3	https://www.peprotech.com/gb/recombinant-human-scd40-ligand
MMP13	0.7	-0.2	-1.2	54	Matrix metallopeptidase 13 - Wikipedia
CD30/TNFRSF8	0.7	-0.2	1.7	63.7	https://www.uniprot.org/uniprotkb/P28908/entry
Arginase1	0.4	-1.2	0.6	34.7	https://www.uniprot.org/uniprotkb/P05089/entry
IL15	-0.1	1.5	1.2	12.9	https://www.peprotech.com/gb/recombinant-human-il-15
CD73	0.5	-1.9	2.9	63.4	https://www.uniprot.org/uniprotkb/P21589/entry
FLT3L	-0.1	-1.1	1.5	17.6	https://www.peprotech.com/gb/recombinant-human-flt3-ligand
IFNα2	-0.1	-1.8	1.2	21.6	https://www.uniprot.org/uniprotkb/P01563/entry
IL12p70	0.2	-0.7	0.5	75	https://www.peprotech.com/gb/recombinant-human-il-12-p70
FGF2	-0.3	0.9	1.4	30.8	https://www.uniprot.org/uniprotkb/P09038/entry
IL12p40	-0.8	-0.7	1.2	37.2	https://www.uniprot.org/uniprotkb/P29460/entry

Supplementary Table 4. Expression of CD25 and CCR4 on CD4+ pleural infiltrating T cells. These data are excerpted from a project to determine the cell surface proteome of different cell populations within MPE cells (Donnenberg *et al*., Immune Landscape of Malignant Pleural Effusions and Peritoneal Ascites. Presented at CYTO 2018, Prague Czech Republic). MPE cells were first stained in bulk with a backbone panel, containing anti-CD45-PE-Cy5.5, anti-CD3-APC-Cy7 and anti-CD4-BV711. Bulk-stained cells were then divided to a 96-well plate containing FITC-, PE-, and APC-conjugated antibodies specific to 228 cell surface proteins (BP80394, BDT FACSCAP Lyoplate; BD Biosciences), one well of which (well E12) contained anti-CD25-APC and one (F08) contained anti-CCR4-Alexa647. Results are expressed as Percent CD25+ or CCR4 cells among CD4+ cells. Regulatory T cells are a Foxp3+ subset of CD25+ CD4+ T cells, so the proportion of functional Regulatory T cells is lower than the results presented here. Pleural CD4+ T cells (and therefore pleural T-reg) did not express CCR4.

BrCA = Breast Cancer; NSCLC = Non-Small Cell Lung Cancer.

**Sample**	**Diagnosis**	**CD25+ among CD3+/CD4+ (%)**	**CCR4+ among CD3+/CD4+ (%)**
MPE083	BrCA (ER-PR-Her2-)	3.66%	0.05%
MPE107	BrCA (ER+PR-Her2+)	0.04%	0.00%
MPE111	BrCA (ER-Her2-)	0.00%	0.00%
MPE128	BrCA	0.40%	0.00%
MPE138	BrCA (ER-Her2-)	0.00%	0.00%
MPE029	NSCLC	1.49%	0.10%
MPE066	NSCLC	0.01%	0.10%
MPE067	NSCLC	0.00%	0.00%
MPE104	NSCLC	0.00%	0.00%
MPE108	NSCLC (Primary with effusion)	2.67%	0.02%
MPE136	NSCLC (Primary with effusion)	0.00%	0.00%
**Mean**		**0.75%**	**0.02%**
**SD**		**1.29%**	**0.04%**
**N**		**11**	**11**
**SEM**		**0.39%**	**0.01%**
